# Qualitative networks: a symbolic approach to analyze biological signaling networks

**DOI:** 10.1186/1752-0509-1-4

**Published:** 2007-01-08

**Authors:** Marc A Schaub, Thomas A Henzinger, Jasmin Fisher

**Affiliations:** 1 School of Computer and Communication Sciences EPFL, 1015 Lausanne, Switzerland

## Abstract

**Background:**

A central goal of Systems Biology is to model and analyze biological signaling pathways that interact with one another to form complex networks. Here we introduce *Qualitative networks*, an extension of *Boolean networks*. With this framework, we use formal verification methods to check whether a model is consistent with the laboratory experimental observations on which it is based. If the model does not conform to the data, we suggest a revised model and the new hypotheses are tested in-silico.

**Results:**

We consider networks in which elements range over a small finite domain allowing more flexibility than Boolean values, and add target functions that allow to model a rich set of behaviors. We propose a symbolic algorithm for analyzing the steady state of these networks, allowing us to scale up to a system consisting of 144 elements and state spaces of approximately 10^86 ^states. We illustrate the usefulness of this approach through a model of the interaction between the Notch and the Wnt signaling pathways in mammalian skin, and its extensive analysis.

**Conclusion:**

We introduce an approach for constructing computational models of biological systems that extends the framework of Boolean networks and uses formal verification methods for the analysis of the model. This approach can scale to multicellular models of complex pathways, and is therefore a useful tool for the analysis of complex biological systems. The hypotheses formulated during in-silico testing suggest new avenues to explore experimentally. Hence, this approach has the potential to efficiently complement experimental studies in biology.

## Background

The emerging field of Systems Biology aims to gain high-level understanding of complex biological systems by studying the structure and dynamics of cellular functions [1,2]. The construction and analysis of models describing biological processes are at the core of this new approach. Computer science provides methods for simulating and analyzing these models. This allows performing *in silico *experiments that lead to new predictions and thus complement traditional experimental approaches. Considering biology from a systems point of view also allows the use of methods designed for the engineering of artificial systems. A wide array of formalisms originating from computer science have already been used to model biological systems. These include process calculi [3], Statecharts and Live Sequence Charts [4-6], variants of Petri nets [7-9], and hybrid models [10,11]. Recently, the term *executable biology *has been proposed for models that consist of an algorithm which mimics the behavior of a studied biological system [12].

Executable models of biological systems can be analyzed using formal verification methods, which were originally designed for the validation of computer systems. Two possible approaches are *testing *and *model checking*. Model checking [13] consists of an exhaustive exploration of the state-space of a given system in order to verify that it always adheres to a set of requirements. In the case of biological systems, requirements are derived from experimental observations. The consistency of a putative executable model can thus be tested with respect to the data on which the model is based [14] (cf. [6] where this method has led to new predictions about vulval development of *Caenorhabditis elegans*, [15-17] where it is used for the analysis of biochemical networks, and [18,19] for the analysis of probabilistic models).

In order to construct executable models of biological pathways, it is necessary to precisely define the interactions between biochemical components. One of the widely used formalism for representing interactions in biology are Boolean networks [20,21]. A Boolean network consists of a set of Boolean variables representing the activity of biochemical components such as proteins or genes. A Boolean function is associated to every component. This function updates the value of the corresponding component at every step of the execution of a Boolean network. In the context of biological pathways, a Boolean network can be built from a graph representing biochemical interactions. Every node of this graph represents a biochemical component. The arcs of the graph are used to represent the interactions between components. Their weight can be positive (for activation) or negative (for inhibition). This graph is used to define Boolean functions for every component. The function sets the value of a component to *true *if the amount of activation is higher than the amount of inhibition. Else the value of the component is set to *false*. Boolean networks therefore provide a highly abstracted view of protein activity or gene expression. This level of abstraction often corresponds to the available qualitative experimental data. Furthermore, the interactions between components are represented in a very similar way in the diagrammatic models that are widely used by biologists. Boolean networks therefore appear to be well suited for the qualitative analysis of biological processes. They have been used recently for modeling a wide array of biological systems, such as the *Arabidopsis thaliana *flower morphogenesis [22], the influence of physical stimuli on cell fate [23], the segment polarity gene network in *Drosophila Melanogaster *[24] and the yeast cell cycle [25]. Boolean networks have been extended in several ways, including probabilistic Boolean networks [26] and the so-called *generalized logical approach *using multivalued levels of concentration [27]. The generalized logical approach has been combined with formal verification methods [16], and constraint programming was used for finding steady states of large networks [28].

In this work, we propose an extension of Boolean networks called *Qualitative networks*. We use this framework to construct an executable model which is analyzed using formal verification methods. We introduce two changes to the Boolean networks framework. First, we use discrete variables instead of Boolean variables for representing gene expression and protein activity levels. Second, we capture a richer set of interactions between components than only activation and inhibition. Any function of the current state of the system can be used to define the target towards which a component will move at the next step. At each step, the level of a component changes towards this target by at most one level. We use simulation and model checking to verify that the implementation of the Qualitative network adheres to a set of specifications derived from the laboratory experimental observations. If necessary, we can incorporate new hypotheses into the model (iterative improvement). Once these are tested in-silico, they should also be validated experimentally. We propose that the stable states of the biological system correspond to the infinitely visited states of the executable model. We introduce an efficient symbolic algorithm for verifying if all infinitely visited states of a Qualitative network satisfy the specifications. We consider the performance of this algorithm for models of different size and complexity, and show that it can be used to analyze large and complex Qualitative networks. We illustrate the ability of Qualitative networks to provide a valid qualitative representation of a studied biological system using a model of the incoherent type-1 feed-forward loop in the E. coli *gal *system.

Our approach to the modeling of signaling pathways allows the detection of gaps in the mechanistic understanding of the studied process, and suggests new biochemical interactions at a similar level of abstraction to the one observed in diagrammatic models. We illustrate this approach by a multicellular model of the interaction between the Notch and the Wnt pathways in mammalian skin cells.

## Results

### Qualitative networks

In this study we propose an approach for constructing executable models of signaling pathways which is based on Qualitative networks, an extension of Boolean networks. In this section, we first explain how biological pathways can be modeled using Boolean networks and then define Qualitative networks. Interactions in biological pathways can be represented as an edge-weighted interaction graph *G *(*V*, *E*). A node *v*_*i *_∈ *V *represents a biological component such as a gene or a protein. An edge *e*_*ij *_∈ *E *from node *v*_*i *_to node *v*_*j *_has a weight *α*_*ij*_, corresponding to the effect of the component represented by *v*_*i *_on the component represented by *v*_*j*_. Activation is specified by a positive value of *α*_*ij*_, inhibition by a negative value. This graph therefore represents the type of interaction between components as well as their strength in a similar way to the diagrammatic models used by biologists. Li *et al*. propose the use of Boolean networks for studying the interactions defined in such a graph [25].

A Boolean network *B *(*C*, *F*) consists of a set of components *C *and a list of boolean functions *F*. Each component c_*i *_∈ *C *has a state, which can take two values: c_*i *_= 1 if the component is active and c_*i *_= 0 if the component is absent or inactive. We use *k *to represent the number of components (*k *= |*C*|). The state *s *of a Boolean network corresponds to the vector (*c*_1_, ..., *c*_*k*_) ∈ {0, 1}^*k *^of the states of all components of the Boolean network. A boolean function *f*_*i *_∈ *F *is assigned to every component. This function maps the current state of the system to a boolean value representing the next state of the corresponding component (*f*_*i *_: {0, 1}^*k *^→ {0,1}). Time in Boolean networks is represented by a discrete variable *t*. We use the notations *c*_*i *_(*t*) and *s *(*t*) to represent the state of a component, respectively the state of the whole system, at time *t*. The value of each protein at the next time step is computed by applying the corresponding boolean function to the current state of the system:

*c*_*i *_(*t *+ 1) = *f*_*i *_(*s *(*t*))     (1)

Given an interaction graph *G *(*V*, *E*), a Boolean network *B *(*C*, *F*) can be obtained as follows: each node of *V *corresponds to a component in *C*. The Boolean function should reproduce the following behavior for every component: if the cumulative activation is higher than the cumulative inhibition, the state of the component is set to active (1) and, vice-versa, if the inhibition is higher than the activation, it is set to inactive (0). Hence, the Boolean function corresponding to each component is defined as follows:

fi(s)={0if∑cj∈sαjicj<01if∑cj∈sαjicj>0ciif∑cj∈sαjicj=0
 MathType@MTEF@5@5@+=feaafiart1ev1aaatCvAUfKttLearuWrP9MDH5MBPbIqV92AaeXatLxBI9gBaebbnrfifHhDYfgasaacH8akY=wiFfYdH8Gipec8Eeeu0xXdbba9frFj0=OqFfea0dXdd9vqai=hGuQ8kuc9pgc9s8qqaq=dirpe0xb9q8qiLsFr0=vr0=vr0dc8meaabaqaciaacaGaaeqabaqabeGadaaakeaacqWGMbGzdaWgaaWcbaGaemyAaKgabeaakiabcIcaOiabdohaZjabcMcaPiabg2da9maaceqabaqbaeqabmGaaaqaaiabicdaWaqaaiabbMgaPjabbAgaMnaaqafabaacciGae8xSde2aaSbaaSqaaiabdQgaQjabdMgaPbqabaGccqWGJbWydaWgaaWcbaGaemOAaOgabeaaaeaacqWGJbWydaWgaaadbaGaemOAaOgabeaaliabgIGiolabdohaZbqab0GaeyyeIuoakiabgYda8iabicdaWaqaaiabigdaXaqaaiabbMgaPjabbAgaMnaaqafabaGae8xSde2aaSbaaSqaaiabdQgaQjabdMgaPbqabaGccqWGJbWydaWgaaWcbaGaemOAaOgabeaaaeaacqWGJbWydaWgaaadbaGaemOAaOgabeaaliabgIGiolabdohaZbqab0GaeyyeIuoakiabg6da+iabicdaWaqaaiabdogaJnaaBaaaleaacqWGPbqAaeqaaaGcbaGaeeyAaKMaeeOzay2aaabuaeaacqWFXoqydaWgaaWcbaGaemOAaOMaemyAaKgabeaakiabdogaJnaaBaaaleaacqWGQbGAaeqaaaqaaiabdogaJnaaBaaameaacqWGQbGAaeqaaSGaeyicI4Saem4CamhabeqdcqGHris5aOGaeyypa0JaeGimaadaaaGaay5Eaaaaaa@759C@

We extend the Boolean network framework by using discrete domains to represent the state of a component and by allowing a rich set of interaction between components. The state of an active component can take several values, rather than being simply *ON*. These values correspond to qualitative levels of gene expression or protein activity, for example, *low, medium, high*. This representation therefore is a middle ground between the Boolean abstraction and quantitative models. The use of discrete domains requires to modify the way the updates of the network are defined. We introduce a *target function *that computes the discrete level towards which a component moves in the next step. The target function is not restricted to modeling activation and inhibition. Every function of the current state of the system can be used as target function. This makes it possible to model interactions that cannot be represented in interaction graphs. One such example is complexation, where the amount of produced protein is bounded by the availability of the proteins required for the interaction. This situation can be modeled by using the minimum of the level of these proteins as a target function. More elaborate target functions can also be used to represent situations in which the effect of a specific activating or inhibiting edge is dependent on the level of additional proteins. We call this extended framework *Qualitative network*. In the following paragraphs, we give a detailed description of Qualitative networks.

A Qualitative network *Q *(*C*, *T*, *N*) consists of a set of components *C *and a list of target functions *T*. The state of a component *c*_*i *_∈ *C *is an integer value between 0 and *N*. These values represent qualitative levels of the components with 0 the lowest and *N *the highest possible level for that component. For each component *c*_*i*_, there exists a target function *target*_*i *_∈ *T *which specifies the level towards which the value of the component should move. This function is computed on the state of the whole system (*target*_*i *_: {0, ..., *N*}^*k *^→ {0, ..., *N*}). At each step, the value of the state of a component can only increase or decrease by a single level. The value of each component at the next time step is therefore computed as follows:

ci(t+1)={ci(t)−1if targeti(S(t))<ci(t)ci(t)+1if targeti(S(t))>ci(t)ci(t)if targeti(S(t))=ci(t)     (2)
 MathType@MTEF@5@5@+=feaafiart1ev1aaatCvAUfKttLearuWrP9MDH5MBPbIqV92AaeXatLxBI9gBaebbnrfifHhDYfgasaacH8akY=wiFfYdH8Gipec8Eeeu0xXdbba9frFj0=OqFfea0dXdd9vqai=hGuQ8kuc9pgc9s8qqaq=dirpe0xb9q8qiLsFr0=vr0=vr0dc8meaabaqaciaacaGaaeqabaqabeGadaaakeaacqWGJbWydaWgaaWcbaGaemyAaKgabeaakiabcIcaOiabdsha0jabgUcaRiabigdaXiabcMcaPiabg2da9maaceqabaqbaeaabmGaaaqaaiabdogaJnaaBaaaleaacqWGPbqAaeqaaOGaeiikaGIaemiDaqNaeiykaKIaeyOeI0IaeGymaedabaGaeeyAaKMaeeOzayMaeeiiaaIaemiDaqhcbiGae8xyaeMae8NCaiNae83zaCMaemyzauMaemiDaq3aaSbaaSqaaiabdMgaPbqabaGccqGGOaakcqWGtbWucqGGOaakcqWG0baDcqGGPaqkcqGGPaqkcqGH8aapcqWGJbWydaWgaaWcbaGaemyAaKgabeaakiabcIcaOiabdsha0jabcMcaPaqaaiabdogaJnaaBaaaleaacqWGPbqAaeqaaOGaeiikaGIaemiDaqNaeiykaKIaey4kaSIaeGymaedabaGaeeyAaKMaeeOzayMaeeiiaaIaemiDaqNae8xyaeMae8NCaiNae83zaCMaemyzauMaemiDaq3aaSbaaSqaaiabdMgaPbqabaGccqGGOaakcqWGtbWucqGGOaakcqWG0baDcqGGPaqkcqGGPaqkcqGH+aGpcqWGJbWydaWgaaWcbaGaemyAaKgabeaakiabcIcaOiabdsha0jabcMcaPaqaaiabdogaJnaaBaaaleaacqWGPbqAaeqaaOGaeiikaGIaemiDaqNaeiykaKcabaGaeeyAaKMaeeOzayMaeeiiaaIaemiDaqNae8xyaeMae8NCaiNae83zaCMaemyzauMaemiDaq3aaSbaaSqaaiabdMgaPbqabaGccqGGOaakcqWGtbWucqGGOaakcqWG0baDcqGGPaqkcqGGPaqkcqGH9aqpcqWGJbWydaWgaaWcbaGaemyAaKgabeaakiabcIcaOiabdsha0jabcMcaPaaaaiaawUhaaiaaxMaacaWLjaWaaeWaaeaacqaIYaGmaiaawIcacaGLPaaaaaa@9F11@

When *N *= 1, this update function is equivalent to the update function of Boolean networks (Equation 1). It follows that Qualitative networks are a generalization of Boolean networks: the Qualitative network *Q *(*C*, *F*, 1) is equivalent to the Boolean network *B *(*C*, *F*).

A particular kind of target function is directly obtained from the qualitative graph describing the studied biological system. It is used to model activation and inhibition in a similar way to the one describe for Boolean networks. In Boolean networks, the sum of all contributions was sufficient to decide on the next value of a component. Here we need to obtain a target function that ranges over {0, ..., *N*}, and for which intermediate values are meaningful. In order to obtain the target function for component *c*_*i*_, we compute separately the amount of activation *act*_*i *_and the amount of inhibition *inh*_*i *_sensed by this component. Both of these values are scaled so that they are proportional to the weighted amount of activation, respectively inhibition. When all activating components are at their highest value, then *act*_*i *_= *N*. Similarly, when all inhibiting components are at their highest value then *inh*_*i *_= *N*.

acti(s)=∑αji>0αjicj∑αji>0αjiinhi(s)=∑αji<0αjicj∑αji<0αji
 MathType@MTEF@5@5@+=feaafiart1ev1aaatCvAUfKttLearuWrP9MDH5MBPbIqV92AaeXatLxBI9gBaebbnrfifHhDYfgasaacH8akY=wiFfYdH8Gipec8Eeeu0xXdbba9frFj0=OqFfea0dXdd9vqai=hGuQ8kuc9pgc9s8qqaq=dirpe0xb9q8qiLsFr0=vr0=vr0dc8meaabaqaciaacaGaaeqabaqabeGadaaakqaabeqaaiabdggaHjabdogaJjabdsha0naaBaaaleaacqWGPbqAaeqaaOGaeiikaGIaem4CamNaeiykaKIaeyypa0ZaaSaaaeaadaaeqbqaaGGaciab=f7aHnaaBaaaleaacqWGQbGAcqWGPbqAaeqaaOGaem4yam2aaSbaaSqaaiabdQgaQbqabaaabaGae8xSde2aaSbaaWqaaiabdQgaQjabdMgaPbqabaWccqGH+aGpcqaIWaamaeqaniabggHiLdaakeaadaaeqbqaaiab=f7aHnaaBaaaleaacqWGQbGAcqWGPbqAaeqaaaqaaiab=f7aHnaaBaaameaacqWGQbGAcqWGPbqAaeqaaSGaeyOpa4JaeGimaadabeqdcqGHris5aaaaaOqaaiabdMgaPjabd6gaUjabdIgaOnaaBaaaleaacqWGPbqAaeqaaOGaeiikaGIaem4CamNaeiykaKIaeyypa0ZaaSaaaeaadaaeqbqaaiab=f7aHnaaBaaaleaacqWGQbGAcqWGPbqAaeqaaOGaem4yam2aaSbaaSqaaiabdQgaQbqabaaabaGae8xSde2aaSbaaWqaaiabdQgaQjabdMgaPbqabaWccqGH8aapcqaIWaamaeqaniabggHiLdaakeaadaaeqbqaaiab=f7aHnaaBaaaleaacqWGQbGAcqWGPbqAaeqaaaqaaiab=f7aHnaaBaaameaacqWGQbGAcqWGPbqAaeqaaSGaeyipaWJaeGimaadabeqdcqGHris5aaaaaaaa@7AA5@

The target function is the difference between the amount of activation and the amount of inhibition. When there is no inhibition, the output of the target function corresponds to the amount of activation. The level of the component will not exceed the amount of the activation. When the amount of inhibition is higher than the amount of activation, the value of the target function is zero. We need to consider a special case when all incoming edges of a component are inhibitions. In this case, we must assume that the mechanism leading to the presence of this component is not modeled, and that, in the absence of inhibition, the target function of this variable will return the maximal value. We obtain the following definition of the target function:

targeti(s)={max⁡(0,acti(s)−inhi(s))if max(αji)>0N−inhi(s)if max(αji)≤0     (3)
 MathType@MTEF@5@5@+=feaafiart1ev1aaatCvAUfKttLearuWrP9MDH5MBPbIqV92AaeXatLxBI9gBaebbnrfifHhDYfgasaacH8akY=wiFfYdH8Gipec8Eeeu0xXdbba9frFj0=OqFfea0dXdd9vqai=hGuQ8kuc9pgc9s8qqaq=dirpe0xb9q8qiLsFr0=vr0=vr0dc8meaabaqaciaacaGaaeqabaqabeGadaaakeaacqWG0baDieGacqWFHbqycqWFYbGCcqWFNbWzcqWGLbqzcqWG0baDdaWgaaWcbaGaemyAaKgabeaakiabcIcaOiabdohaZjabcMcaPiabg2da9maaceqabaqbaeaabiGaaaqaaiGbc2gaTjabcggaHjabcIha4jabcIcaOiabicdaWiabcYcaSiabdggaHjabdogaJjabdsha0naaBaaaleaacqWGPbqAaeqaaOGaeiikaGIaem4CamNaeiykaKIaeyOeI0IaemyAaKMaemOBa4MaemiAaG2aaSbaaSqaaiabdMgaPbqabaGccqGGOaakcqWGZbWCcqGGPaqkcqGGPaqkaeaacqqGPbqAcqqGMbGzcqqGGaaicqWFTbqBcqWFHbqycqWF4baEcqGGOaakiiGacqGFXoqydaWgaaWcbaGaemOAaOMaemyAaKgabeaakiabcMcaPiabg6da+iabicdaWaqaaiabd6eaojabgkHiTiabdMgaPjabd6gaUjabdIgaOnaaBaaaleaacqWGPbqAaeqaaOGaeiikaGIaem4CamNaeiykaKcabaGaeeyAaKMaeeOzayMaeeiiaaIae8xBa0Mae8xyaeMae8hEaGNaeiikaGIae4xSde2aaSbaaSqaaiabdQgaQjabdMgaPbqabaGccqGGPaqkcqGHKjYOcqaIWaamaaaacaGL7baacaWLjaGaaCzcamaabmaabaGaeG4mamdacaGLOaGaayzkaaaaaa@84BD@

We use Qualitative networks to define executable models of biological systems. The list of target functions specifies how the different molecules in the system influence each other. When creating a model based on the mechanistic understanding of a biological process, we use this information to define a set of target functions. Where necessary, we separate the representation of protein activity from the expression level of the corresponding gene. This is useful for modeling cases in which there are interactions at the transcription level as well as protein-protein interactions. During the analysis process, we may modify the definition of the target functions. While Qualitative networks allow for any kind of target function, we use a subset of functions that represent specific biological interactions, such as the activation/inhibition function described above. This ensures that new hypothesized interactions between components are defined at a level where they have a clear biological meaning. The implementation of models defined as Qualitative networks is described in the *Methods *section.

### Iterative model improvement

A model in general, and an executable model in particular, is meant to represent how the system that we study actually works. Thus, it is natural to expect that the model would be consistent with the data obtained from laboratory experimentation with this system. We use *simulation *and *model checking *[13], which allows to formally check all the different executions of the model against a formal specification. By exploring all the possible states and transitions of the model we can determine whether some requirement holds for the model. In the case that the property does not hold, the model-checking algorithm supplies a *counter example*: an execution of the system that does not satisfy a given requirement. We use the information provided by this counter example to suggest modifications of the Qualitative network representing the studied system.

**Figure 1:**
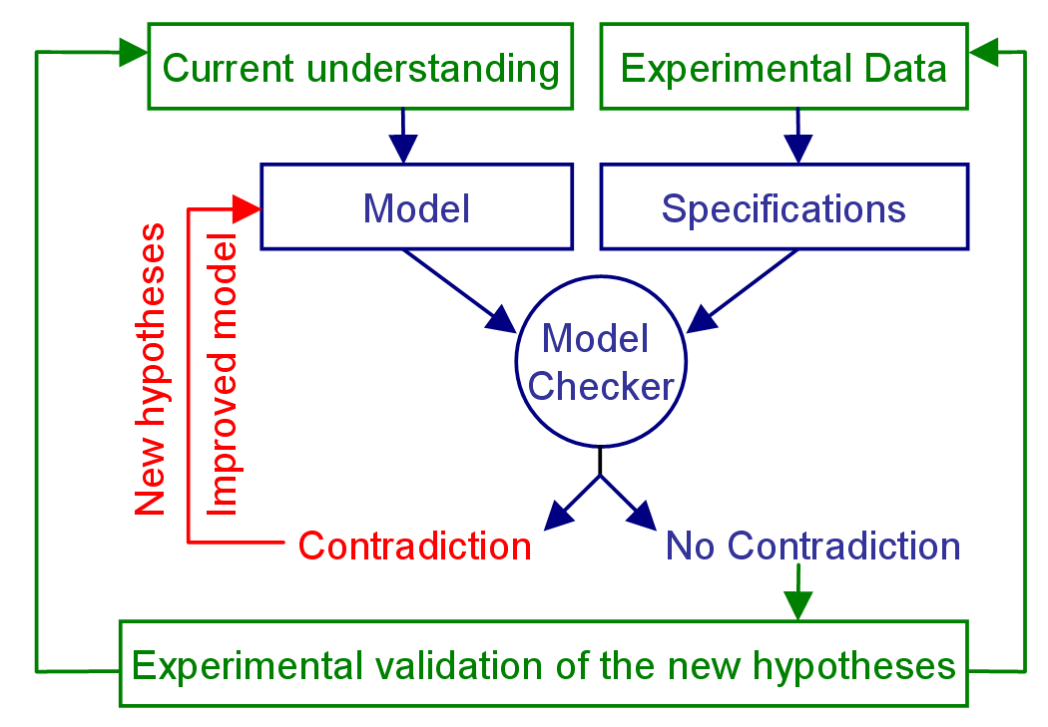
**Iterative improvement of the model**. Schematic view of the iterative improvement process used to build a model which is consistent with the experimental data. The verification process is represented in blue. The improvement of the model based on counter examples is represented in red. The outer improvement loop, based on laboratory experiments, is represented in green.

Our methodology in using Qualitative networks is the *iterative improvement process *(Figure 1). We stress that the usage of Qualitative networks is not restricted to this methodology. The advantages of multiple values, flexible target functions, and the analysis algorithms suggested below, can be used regardless of the modeling methodology chosen by the user.

The iterative improvement process consists of the following. We first derive a set of specifications from prior laboratory experimental results. We build an initial putative model based on the current mechanistic understanding of the studied biological process and the working hypotheses we would like to evaluate. We then use formal verification methods to verify if the proposed model conforms with the specification. If the model fails this test, we deduce that the model does not conform to the data. We use counter examples, to suggest a revised model by making new hypotheses. The new model is then evaluated again, and these steps are repeated until no contradiction is found between the model and the specifications. At this point, we might have had to modify our hypotheses, both in discarding unverifiable assumptions and in adding assumptions that are necessary for the model to comply with the specifications. The resulting model, and the corresponding new set of hypotheses, are then known to be consistent with the experimental results. At this stage, it is possible to query the model to gain additional knowledge about the biological system. This can be done either by modifying the model to evaluate or by adding specifications that could provide additional information on the behavior of the system. Hypotheses and changes in the model leading to interesting behaviors are candidates for experimental validation. The result of such an experiment, whether confirming the information gained from the computational analysis or not, can then be added to the specification. This creates a second, outer, improvement loop which combines computational and experimental validation.

The specifications derived from laboratory experimental results can be separated into two categories. *Low-level specifications *involve changes in the level of protein activity or gene expression (e.g. over-expression of a given protein leads to a decrease of another protein). *High-level specifications *consider changes at the cellular level, such as cell fate or susceptibility to carcinogenesis. Since we can only observe the level of individual proteins, we need to define how these relate to changes at the cellular level. Experimental results are often related to manipulation, such as gene over-expression or knockout. These manipulation lead to changes in the behavior of the model. The *Methods *section provides additional details on how specifications are defined and verified.

This iterative improvement approach can be applied to highly non-deterministic models, hence the use of model checking. Qualitative networks, like Boolean networks, have a deterministic behavior, and it is therefore sufficient to perform one execution per initial state, and verify if each of these executions satisfies the specifications. If some executions do satisfy the specifications, but others do not, then the model is not constrained enough, and we need to formulate hypotheses that allow avoiding the executions which do not satisfy the specifications. Ideally, the new hypotheses would not contradict the current mechanistic understanding of the system, but rather suggest additional putative interactions between components. If there is a specification which is not satisfied by any execution of the model, then there is a contradiction between the mechanistic understanding of the system and the experimental data. In this case, the modifications needed for the model to satisfy the requirement will suggest alternative mechanistic models of the studied system that are more consistent with the experimental data.

In the following sections, we describe two additional methods for analyzing the model during the improvement process: the use of non-determinism on parts of the model, and the efficient computation of all infinitely visited states.

### Use of non-determinism

In the case where every interaction in the model is precisely defined, enumerative networks are deterministic. Non-determinism can, however, be used to represent unknown interactions. In particular, rather than studying a complete multicellular model, the model of a single cell can be studied by representing inter-cellular interactions non-deterministically.

The level of proteins that are external to the cell are modeled by non-deterministic variables. These variables cannot jump arbitrarily from one value to another value, but only change by one level per time step. At each time step, the variable can either increase, decrease or remain constant. This is sufficient to capture any possible behavior since updates of components are bounded by Equation 2. The set of possible behaviors of the state of this protein is therefore a superset of the possible behaviors for any possible target function.

Instead of simulation, we use model checking, which explores all possible executions of a non-deterministic system. This leads to the analysis of a superset of the possible executions of any model with more constrained inter-cellular interactions. If no execution of the system satisfies the specification, then it is impossible that any model including this cell can satisfy the specifications, no matter how the inter-cellular interactions are defined. If some executions satisfy the specifications, but other do not, then it is not possible to predict the behavior of the cell with more constrained inter-cellular interactions. This approach is therefore useful for finding out if the improvement should be performed at the level of the inter-cellular communication or at the intracellular level.

### Finding infinitely visited states

We propose an efficient symbolic algorithm for computing the infinitely visited states of a Qualitative network. A state *S *is said to be infinitely visited if there is an execution of the network in which the state appears infinitely often. In a biological context, the infinitely visited states correspond to the stable states of the system. In the context of specification-based analysis of the model, we are therefore interested in knowing if the specification holds on all infinitely visited states. This captures the fact that, as opposed to computerized systems, biological system do not have an initial state, but rather are continuously behaving. After cell division, for example, the two resulting cells would have proteins levels that are at a qualitative level similar to the levels in the original cell. States that are not infinitely visited can thus be considered as unstable states. If the model is in such a state, it will evolve towards an infinitely visited state in a bounded number of steps, and then remain in infinitely visited states for an indefinite time. Therefore, we do not insist that the specification holds on states that are not infinitely visited.

Analysis of Boolean networks also include studies of the attractors of the model. In discrete, deterministic models, attractors are loops of one or more states that are visited infinitely often. Questions of interest include: does the system end up in one or several attractors, and what initial conditions lead to which attractor. This analysis requires exploring the executions starting from all initial states. The number of possible initial states is exponential in the number of variables, and therefore we try to prune them by using the available information on the system. In case that no biologically meaningful definition of the set of initial state exists, it is necessary to consider all states as being initial. In this case, enumerative exploration is practically intractable even for relatively small models. In the *Methods *section, we describe a symbolic algorithm for finding all the attractors of a model, based on compact representation of sets of states using Binary Decision Diagrams (BDDs) [29].

The algorithm we propose uses the structure of multicellular Qualitative networks by interleaving composition and computation of infinitely visited states. We first consider a partial model consisting only of the first cell. The behavior of components in neighbor cells are abstracted by allowing them to change non-deterministically. We compute the set of infinitely visited states of this partial model. This set contains the projection of any infinitely visited state of the complete model onto the variables of the partial model. We use this information to compute the infinitely visited states of a partial model consisting of the first two cells. This process is repeated to obtain the complete model and the set of infinitely visited states. We further optimize this algorithm by using *partitioned transition relations *[30]. Based on the target functions list of the Qualitative network, we define a transition relation for a single cell. We then use it repeatedly to compute the next state of the multicellular system, rather than using a single transition relation for the whole model. The *Methods *section contains a detailed description of this algorithm.

### Modeling network motifs using Qualitative networks

Transcription regulation networks contain patterns that occur significantly more often in a real network than in a random network with the same characteristics. These patterns are called network motifs [31]. The multiple values used by Qualitative networks may be required to accurately model the qualitative behavior of common network motifs in a way that would be difficult, if not impossible, to achieve with Boolean values. We illustrate this ability by considering the case of the incoherent type-1 feed-forward loop (I1-FFL) in *E. coli*.

In this motif, transcription factor *X *activates transcription factor *Y *and gene *Z*, and transcription factor *Y *represses *Z*. The response time is defined as the time needed for *Z *to increase halfway to its steady-state level. Compared to simple regulation (only *X *activates *Z*), the I1-FFL has a shorter response time to the activation of *X*. When *X *is activated, the level of both *Y *and *Z *start to increase. Once the level of *Y *exceeds the repression threshold for the *Z *promoter, it will start repressing *Z*. As a consequence, the level of *Z *starts decreasing towards a low steady-state level. The response time is thus shorter, because the steady-state level of *Z *is lower than for simple activation, but its initial transcription rate is the same in both cases. This behavior has been observed both using quantitative models [32] and experimentally in the *gal *system in *E. coli *[33].

**Figure 2:**
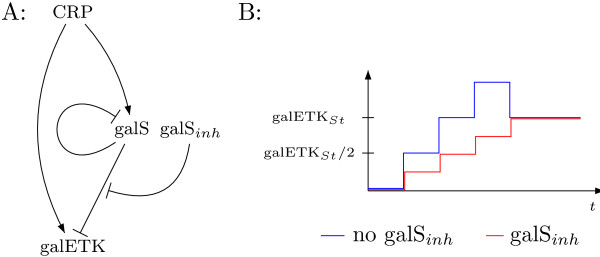
**Model of the gal system in E. coli**. *A*: View of the *gal *system in E. coli. The activator *CRP *activates both *galETK *and *GalS. GalS *inhibits *galETK *and negatively auto-regulates itself. *GalS*_*inh*_, which is a shortcut representing both *β-D-galactose *and *D-fucose*, cancels the inhibitory effect of *Gals*_*S *_on *galETK*. *B*: Evolution of the level of *galETK *when *CRP *is activated at time *t *= 0. The level are represented using relative values with respect to the stable state *galETK*_*St*_. The situation in which no *GalS*_*inh *_is not expressed is represented in blue. This corresponds to an I1-FFL. The situation in which *GalS*_*inh *_is present is represented in red. In this situation, the model behaves like with a simple activation. The response time, measured as the time needed to reach *galETK*_*St*_/2 is shorter in the absence of *GalS*_*inh*_.

In the *gal *system (Figure 2a), an I1-FFL links the activator *CRP *to the galactose utilization operon *galETK*. When there is glucose starvation, *CRP *gets activated. *CRP *then activates both *galETK *and *GalS*, and *GalS *inhibits *galETK*, hence the I1-FFL motif. In addition, *β-D-galactose *or *D-fucose *(henceforth called *GalS*_*inh*_) unbind *GalS *from the *galETK *promoter region, thus cancelling its inhibitory effect. Finally, *GalS *negatively auto-regulates its own expression. We construct a Qualitative network model of this system based on these interactions. This model needs to satisfy two requirements to be realistic. First, the activation of *CRP *must lead to the activation of *galETK *independently of the level of *GalS*_*inh*_. Second, the time needed by *galETK *to increase halfway to its steady-state needs to be shorter when *GalS*_*inh *_is absent than when it is present. Figure 2b shows the evolution over time of the level of *galETK *after *CRP *is activated for both case. This result is qualitatively similar to the experimental measurements of the *galETK *expression performed by Mangan *et al*. [33].

Qualitative networks are able to provide realistic qualitative approximations of the behavior of further common network motifs, such as other types of feed-forward loops and regulated-feedback motifs that appear in developmental transcription networks [34].

### Case study: a model of the interaction between the Notch and Wnt signaling pathways

We apply our modeling approach to a model representing the crosstalk between the Notch and Wnt pathways in mammalian keratinocytes. Both pathways play a key role in the control of cell proliferation and differentiation and have been linked to several types of cancer. We first present the biological background, then introduce the Qualitative network model for a single cell and for a model with five cell representing the multiple layers of the mammalian epidermis. We define a set of specifications derived from laboratory experimental perturbations on these pathways, and use them to analyze the possible executions of the model.

#### Crosstalk between the Notch and Wnt signaling pathways

Notch signaling plays a key role in the control of the development of various tissues, with the particularity that, depending on the context, it can either induce differentiation or maintain cells in a proliferating state (role in carcinogenesis reviewed in [35]). The Notch receptor protein is located across the cell membrane and is composed of two parts – an extracellular component and a cytoplasmic part. The extracellular component of Notch can bind to a ligand expressed on the membrane of a neighboring cell. In mammals, there are four types of Notch proteins, three types of Delta-like ligands and two types of Serrate-like ligands, which are called Jagged. The binding of a ligand to the Notch receptor leads to a proteolytic cleavage of the two parts of the Notch protein, thus releasing its cytoplasmic part – the Notch intracellular domain (Notch-IC). Notch-IC then travels to the nucleus, where it binds to the transcription factor CSL. In the absence of Notch-IC, CSL acts as a transcription inhibitor, but the presence of Notch-IC converts it to a transcription activator, thus leading to the expression of its transcriptional targets.

The Wnt signaling pathway has a critical regulating role in stem cells, and has been associated with cancer in several tissues (reviewed in [36]). The central player in the canonical Wnt signaling pathway (reviewed in [37]) is the protein *β*-Catenin. In the absence of Wnt signaling, the newly formed *β*-Catenin is found in a degradation complex composed of the proteins adenomous polyposis coli (APC), the scaffolding protein Axin and the glycogen-synthase kinase-3*β *(GSK3*β*). The GSK3*β *phosphorilates *β*-Catenin, which is then degradated by proteasomes. Wnt is a short range signaling protein which interacts with a serpentine receptor of the Frizzled family. The consequence of this interaction are not completely understood, but it appears that an axin-binding molecule, Dishevelled (DSH), gets phosphorylated and starts inhibiting the degradation of *β*-Catenin. This *β*-Catenin can therefore accumulate in the cytoplasm and travel to the nucleus, where it interacts with the Tcf/Lef DNA-binding proteins. In the absence of *β*-Catenin, a co-repressor like Groucho binds to Tcf/Lef, thus blocking the transcription of its downstream target genes. When interacting with *β*-Catenin, Tcf/Lef becomes a transcription activator, thus leading to the activation of the downstream target genes of the Wnt signaling pathway.

**Figure 3:**
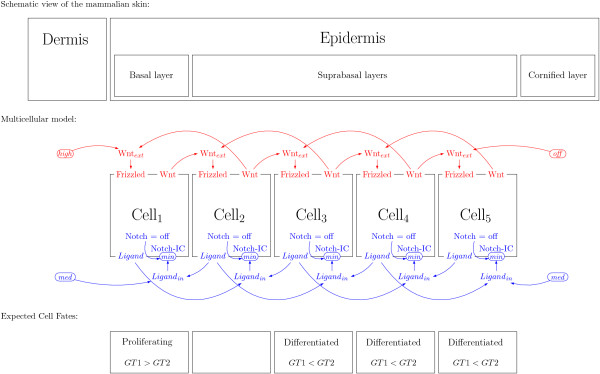
**Schematic view of the multicellular model**. View of the different layers of the mammalian skin, and how they are represented in the multicellular model. Connections through the Wnt pathway are represented in red and connections through the Notch pathway are represented in blue. The level of Notch receptor of each cell is fixed, and the corresponding values are indicated in the cells. Both the dermis and the cornified layer (composed of dead cells) are not represented in the model. The required result for each cell in the case of wild type simulation is indicated below the cell. Cells of the basal layer are proliferating, while cells of the suprabasal layers are differentiated.

The outermost layer of the human skin, the epidermis, can be divided into several layers: the basal layer, which is the closest to the underlying dermis, the suprabasal layer, and the cornified layer, which is mainly composed of dead cells (Figure 3). In normal tissue, most proliferating keratinocytes are located in the basal layer. They migrate to the suprabasal layer when they have initiated terminal differentiation and then stop proliferating. Notch is expressed in the human epidermis, at a higher level in the suprabasal layers than in the basal layer. The Jagged ligand is co-expressed with Notch in the skin and has been proposed as a positive feedback mechanism for sustained Notch signaling triggering differentiation [38]. The interaction between the Notch and the Wnt pathways are involved in the transition from a proliferating to a differentiated state in keratinocytes. *In vitro *experiments have shown that Notch signaling induces terminal differentiation in both murine and human skin [39]. The tissue specific ablation of the Notchl receptor in mouse epidermis leads to hyperplasia in the epidermis, the development of tumors and an increased susceptibility to chemical carcinogens – thus indicating that Notch acts as a tumor suppressor in mammal skin [40]. Notch was suggested to interact with the Wnt pathway through its direct transcriptional target p21, which is a negative transcription regulator of Wnt [41].

#### Model construction

**Figure 4:**
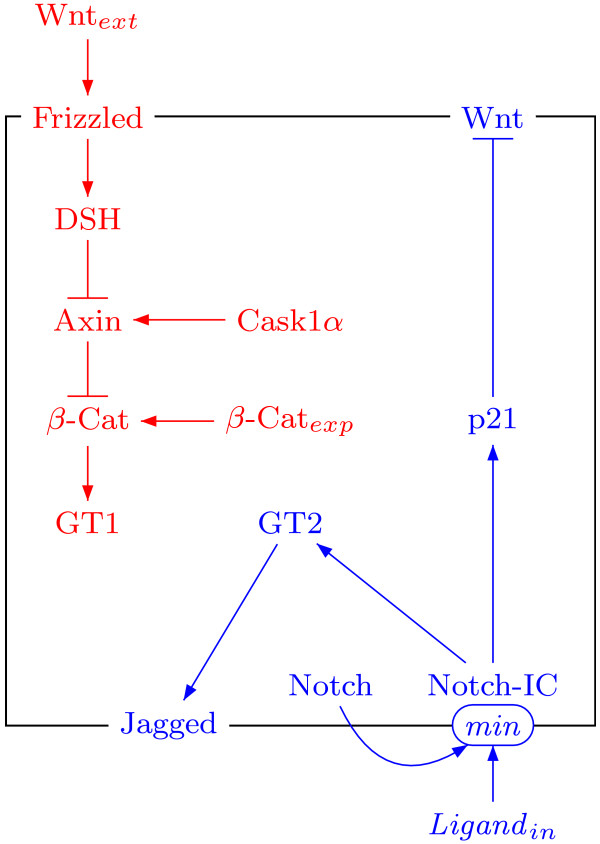
**Visualization of a single cell**. The components of the Wnt pathway are represented in red and the components of the Notch pathway in blue. The canonical Wnt pathway starts from the extracellular level of the short range signaling Wnt protein, which we represent by the *Wnt*_ext _variable. This short range molecule binds to the *Frizzled *receptor, which leads to an increase in the intracellular level of *DSH*. We therefore represent the interaction between these molecules by an activation from Wnt_*ext *_to Frizzled and then to DSH. The level of the scaffolding protein *Axin *is dependent on the level of DSH which acts as an inhibitor and Casein Kinase 1 *α *(represented by the variable *Cask1α*), which is assumed to be an activator. *β-Catenin *is phosphorylated by a complex composed, amongst others, by Axin. We separately represent the expression level of *β*-Catenin, *β-Catenin*_*exp*_. *β*-Catenin is activated by *β*-Catenin_*exp *_and inhibited by Axin. The family of downstream target genes of the canonical Wnt signaling pathway, (*GT1*), are transcriptionally activated by *β*-Catenin. The Notch pathway is based on the cell-cell interaction between the Notch receptor (whose level is represented by the variable *Notch*) and the ligands expressed on the membrane of the immediate neighboring cells (*Ligand*_*in*_). Since a successful binding between the receptor and the ligand leads to the cleavage of the intracellular part of Notch (*Notch-IC*) we use the minimum target function to model this interaction. We represent the level of the downstream target genes of Notch signaling as a single variable (*GT2*), which is activated by Notch-IC. Amongst these targets is the protein *p21*, which is activated by Notch-IC and inhibits *Wnt*. Wnt is an external variable of the cell. The downstream targets of Notch signaling activate the *Jagged *ligand.

We build a model of the Notch and Wnt pathways in the epidermal layer of mammalian skin. Our model is composed of five identical keratinocytes, each of them representing one layer of the epidermis. Protein activity and gene expression are represented by variables that can take four values: *off, low, medium *and *high*. All activation and inhibition interactions have an equal weight (*α*_*ij *_∈ {-1, 0, 1}). Figure 4 represents the two pathways in a single cell. Except for the intracellular part of Notch (*Notch-IC*), the target function of all other components is the difference between activation and inhibition, as defined by Equation 3. The target function of the *Notch-IC *component is the minimum between the level of Notch receptor on the cell membrane and the level of ligand on the membrane of the neighboring cells.

The complete model consists of a single row of five cells, which are named *Cell*_1 _to *Cell*_5_. Neighboring cells are connected both through the Wnt and the Notch pathways (Figure 3). Cell_1_, the leftmost cell on the visualization of the model, represents the lowest layer of keratinocytes of the epidermis, which means that the dermis is to its immediate left. This cell should adopt a proliferating fate. The upper skin, to which the keratinocytes migrate before they die, is represented on the right side of the model. We consider that the two immediate neighbors contribute to the level of ligand sensed by a cell. The level of ligand sensed by a cell (Ligand_*ext*_) is activated by the level of ligand on the membrane of its immediate neighbor, and is thus maximal when the level of ligand in both neighbors is high. The level of Jagged on both extremities of the model is constant at medium. Similarly, we connect the level of Wnt produced by a cell to the level of Wnt sensed by the nearby cells. We consider the case in which a cell does not sense Wnt emitted by itself. In this case we can use the same double activation scheme as for the ligands. On the left side of the model, the level of Wnt is fixed to high since the dermis is known to emit Wnt signaling proteins. In contrast, the level on the right side is fixed to low since Wnt signaling does not occur in the upper layers of the skin.

#### Specifications

In order to be able to define high level specifications, we need to map changes in the level of protein activity to a certain cell fate. We consider the balance between the downstream target genes of Wnt signaling (referred as *Gene Target 1*, or *GT*1) and the downstream target genes of Notch signaling (*Gene Target 2*, or *GT*2). The downstream targets of the Wnt signaling are known to maintain the cells in a proliferating state, whereas the downstream targets of Notch initiate differentiation. Therefore, we make the assumption that a cell in which *GT*1 > *GT*2 is proliferating, whereas a cell in which *GT*1 <* GT*2 is differentiated. Furthermore, since an increase in the level of *GT*1 or a decrease in the level of *GT*2 makes a cell more likely to be proliferating, we assume that this change also makes the cell more sensitive to chemically-induced carcinogenesis. For experiments involving the knockout or the over-expression of a gene, we compare the level of components when the wild type model is in a stable state to the level of the proteins when the altered model reaches a stable state again.

The first high level specification (*H*1) defines the wild type state, and can be split into two parts: Cell_1 _is proliferating (*H*1.1), and Cell_4–5 _are differentiated (*H*1.2).

**H1.1: **GT1 > GT2 in Cell_1_

**H1.2: **GT1 < GT2 in Cell_4–5_

We do not use Cell_2–3 _in these specification, but if a cell has adopted a differentiated fate, cells in a higher sub-layer (on its right in the model) cannot be proliferating. We formulate this as an additional requirement:

**H2.1 **GT1 = GT2 in Cell_*i *_⇒ ∀*j *> *i*, GT1 ≤ GT2 in Cell_*j*_.

**H2.2 **GT1 < GT2 in Cell_*i *_⇒ ∀*j *> *i*, GT1 < GT2 in Cell_*j*_.

We derive further specifications from the experiments performed by Nicolas *et al*. on mouse keratinocytes [40]. They note that Notch knockout leads to increased proliferation, which we translate into a requirement that Cell_4 _is also proliferating.

**H3: **Notch = KO ⇒ GT1 > GT2 in Cell_4_

After several months, mice in which Notch is knocked out begin to develop basal cell-like carcinoma. They are also significantly more sensitive to chemically induced carcinogenesis. In order to relate these long-term changes to variations at the protein level, we assume that the short term effect of the Notch knockout is an increase of GT1 in several cells, or a decrease of GT2 in several cells, or both.

**H4: **Notch = KO ⇒ GT1 increases in Cell_1–5 _or GT2 decreases in Cell_1–5_

The impact of p21 knockout was also considered. Mice with p21 deficiency were more sensitive to chemically induced carcinogenesis, but did not develop tumors on their own. We relate this result to the level of GT1 and GT2 in a similar way to Notch knockout.

**H5: **p21 = KO ⇒ GT1 increases in Cell_1–5 _or GT2 decreases in Cell_1–5_

The work by Nicolas *et.al*. on Mice keratinocytes [40], as well as the work of Devgan *et.al*. [41] on the link between Notch and Wnt through p21, also leads to several low level specifications. These specifications were directly considered when constructing the model and are not detailed here.

#### Analysis of the model

We compute the set of infinitely visited states of the model described above. We obtain a total of 6561 states. All of these states adhere to the specifications *H1 *and *H2*. These states, however, differ as far as the fate of Cell_2 _is concerned. This cell is either proliferating (*GT*1 > *GT*2 in 2916 states), differentiated (*GT*1 <* GT*2 in 3645 states) or in transition from proliferating to differentiated (*GT*1 = *GT*2 in 2916 states). Several of these attractors consist of more than one state, and the relation between *GT*1 and *GT*2 in Cell_2 _can change between the different states of a single attractor. We can therefore conclude that, in this model, Cell_2 _is no longer receiving constant signals for proliferation, but has not yet committed to terminal differentiation. After the model reaches a stable state, modifying it to knockout Notch or p21 allows to verify that the model also satisfies specifications *H3*, respectively *H4*.

Further insights can be gained by studying variations of the model. We consider the hypothesis that Notch-IC activates the transcription of *β*-Catenin, thus partially compensating its inhibitory influence on *β*-Catenin through the Wnt pathway. This hypothesis can be rejected by analyzing a single cell model with non-deterministic levels for both external Wnt and sensed ligand (*Ligand*_*in*_). No execution of this model can satisfy requirement *H*1.1 (*GT*1 > *GT*2) for all the states of the execution that occur infinitely often. This means that in any multicellular model with this interaction, no cell could be proliferating. We can therefore conclude that some other mechanism activating *β*-Catenin must exist. Notch-IC may play a role in the activation of *β*-Catenin, but cannot be the only activating protein.

We also consider the hypothesis that Notch signaling uses the Delta ligand instead of the Jagged ligand. While there is a positive feedback mechanism from Notch-IC to Jagged, Notch-IC inhibits the expression of the Delta ligand on the cell surface. We find that such a model cannot reproduce the wild type behavior (specification *H*1). By analyzing the counterexample traces, we notice that due to the inhibition of the ligand, sustained Notch signaling cannot be achieved in the higher layers of the epidermis. This is consistent with the experimental analysis of the level of Delta in the epidermis showing that Delta is expressed in the basal layer only [42]. In their analysis, Lowell *et al*. describe stem-cell clusters with local concentration of Delta. In order to model and analyze local effect such as cell clusters, it would be necessary to consider significantly larger multidimensional models.

### Performance analysis

In this section, we describe the performance of the symbolic algorithm used to compute all infinitely visited states of a Qualitative network. We first consider variations of the Notch/Wnt model described above. We then use an arbitrary model to show that the method can be applied to models of more complex interactions between components. Finally, we show that our algorithm performs better than simpler symbolic algorithms. The *Methods *section gives additional details about the settings used for these analyses.

The computation of all infinitely visited states of the five-cells Notch/Wnt model takes 21 minutes. This model has 4^60 ^≈ 10^36 ^initial states. This is a significantly larger number than previously studied by Boolean networks (for example, the model used by Li *et al*. [25] has 2^11 ^= 2048 states). In order to evaluate the performance of our symbolic algorithm on larger models, we extend the Notch/Wnt model up to 12 cells. We add cells both to the basal layer (on the left side of the model) and to the suprabasal layer (on the right side of the model). As in the five-cells model, the level of Notch receptor is set to *off *in the basal layer, and to *high *in the suprabasal layer. While the original model has only one cell with an intermediate level of Notch receptor, we introduce a second cell in the larger model in order to obtain a gradient. These models have been developed for the purpose of performance analysis only, and do not provide additional biological insights. Table 1 shows the time needed to compute the set of all infinitely visited states for models with 3 to 12 cells. The results illustrate that we can compute the set of infinitely visited states of a model which has more than 10^86 ^initial states.

**Figure 5:**
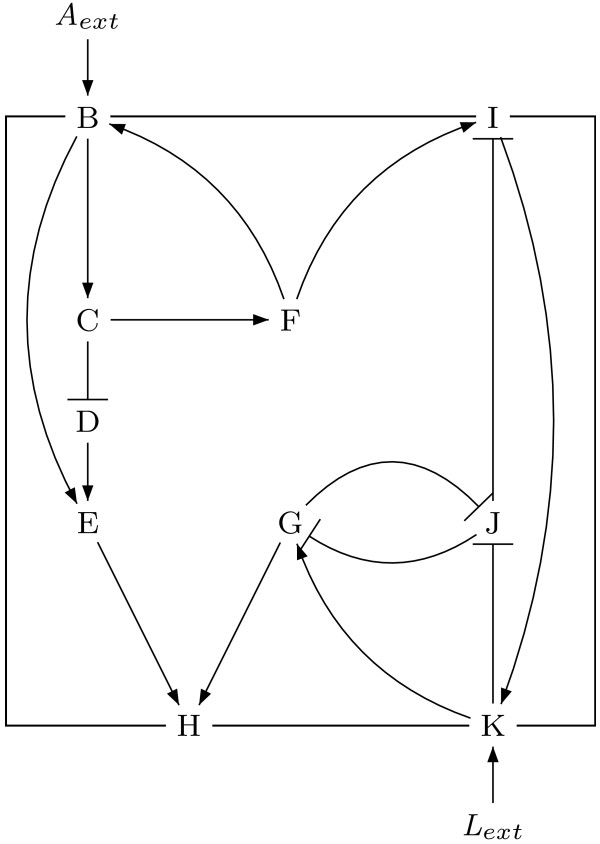
**Arbitrary pathways for performance evaluation**. This cell contains arbitrary chosen pathways that have no biological meaning. The interactions between components in this example were chosen in order to obtain a complex transition function. The component *A*_*ext *_is connected to the component *I *of the neighbor cells. The component *L*_*ext *_is connected to the component *H *of the neighbor cells.

It can be observed that adding a cell in the basal layer has a significantly smaller impact on the performance than adding a cell in the suprabasal layer. This is due to the fact that the possible behaviors of the cells in the basal layer are very limited due to the lack of Notch signaling. In general, the performance of symbolic algorithms is dependent on how compact the representation of the sets of states and the transition relation are. This complexity is directly dependent on the structure of the model. In the case of Qualitative networks, choosing a different combination of target functions has an impact on the performance even though the number of components stays the same. We illustrate this by constructing an arbitrary Qualitative network (Figure 5), which has no biological meaning except that we only use the activation/inhibition target function. This model contains the same number of components per cell as the Notch/Wnt model. The interactions between the components are more complex. In order to compare the complexity of the two models, we consider the size of the BDD used to represent the transition relation of a single cell. This size, measured as the number of nodes in the BDD, relates more or less to the required memory. The arbitrary Qualitative network is represented using 279'504 BDD nodes, compared to 39'564 BDD nodes for the Notch/Wnt model. This increase in complexity is reflected in the performance of the infinitely visited states computation. When considering only three cells this computation takes 33 seconds for the Notch/Wnt model, and 113 minutes for the arbitrary Qualitative network.

Our algorithm interleaves the composition of a multi-cellular model with the computation of infinitely visited states. This approach outperforms symbolic algorithms that directly compute the set of infinitely visited states of the complete model. The application of the direct algorithm to models of the Notch/Wnt pathway with four or more cells fails, because the symbolic representations of the sets of states becomes too large. On the three-cell model, our algorithm needs 33 seconds and the direct algorithm 231 seconds. The advantage of first computing the infinitely visited states on partial models is that the sets of potentially infinitely visited states is smaller than in the direct algorithm. This is illustrated in Table 2, which provides details about the application of our method to the ten-cells Notch/Wnt model. For each partial model, we indicate the initial set of states we start from, as well as the set of infinitely visited states of the partial model after the computation. In particular, it can be noted that the computation of the infinitely visited states of the complete model starts from a set of approximately 10^13 ^states. An algorithm that directly computes all infinitely visited states would start from the set of all states of the system (approximately 10^72^).

The symbolic approach we propose is able to compute the infinitely visited states for large multicellular Qualitative networks and to handle complex models. By tailoring the algorithm specifically for Qualitative networks, we can analyze larger models than by directly computing the infinitely visited states.

## Discussion

In this work, we extend Boolean networks to Qualitative networks; networks that allow variables to take discrete values representing qualitative levels of protein activity or gene expression. By introducing a target function, we allow a rich set of interactions between components. We propose a method for analyzing Qualitative networks, that uses formal verification of specifications derived from experimental observations for iteratively improving the model. In order to efficiently analyze the properties of infinitely visited states of large models, we introduce a symbolic algorithm which builds on the structure of our modeling method. We apply this method to build and analyze a multicellular model of the interactions between the Wnt and Notch pathways in mammalian keratinocytes. We show that the proposed symbolic algorithm can be applied to large and complex multicellular models.

### Model analysis approach

Our modeling approach is aimed at using formal verification methods for the analysis of signaling networks in an iterative improvement process which is based on specifications that are directly derived from experimental results. This work therefore combines formal verification methods with the widely used formalism of Boolean network, and its extension to qualitative domains. Since Boolean networks are a particular case of Qualitative networks, the iterative improvement approach can be directly applied to Boolean networks as well.

Our method allows to build a model at a level of abstraction that is similar to the one commonly used in cell biology, and to analyze it using specifications that are directly derived from experimental studies. This approach is therefore useful for biologists to identify gaps between the hypothesized mechanistic description of a pathway of interest, and the experimental data on which the hypothesis has been formulated. Furthermore, the careful analysis of counter examples found using this approach can lead to new hypotheses on how to reconcile the model with the experimental data. We illustrate the usefulness of this approach by an example showing that we can infer a known interaction from the analysis of a model in which this interaction was purposefully ignored. It is known that in mammalian keratinocytes, Notch activates the expression of its ligand Jagged. For the purpose of this example, we ignore that information, and consider that Notch inhibits the expression of the Delta ligand, as it is the case in Drosophila when building our original model. Such a model is not able to reproduce the wild type specification (as formulated in the results section), and we have thus identified a gap between the hypothesized model and the experimental data. The counter example traces we obtain indicate that the model is unable to reproduce the sustained Notch signaling that is necessary in order to reproduce the observed wild type behavior. This strongly suggests that the type of interaction between Notch and its ligand in keratinocytes needs to be changed from inhibition to activation. Applying this change to the model and then running the verification algorithm again shows that the modified model is consistent with all specifications. This means that our refinement process allows us to infer the hypothesis that was left out in this example. Furthermore, a similar approach, although using a different formalism, has lead to new biological insights about the vulval development in *C. elegans *[6].

We believe that the possibility of *in-silico *execution of putative models is an efficient way to help biologists understand the behavior of a complex signaling networks. Counter example traces are helpful not only to detect that a model does not behave as expected, but also to provide a concrete basis on which one can reason about why this is indeed the case. In addition, by querying the model, interesting executions could suggest new experiments to be tested in the lab. We therefore expect that the application of our modeling methodology to other biological systems will help provide new biological insights.

An area in which our approach appears to be particularly useful is the analysis of cell-cell communication in multicellular models. Given the size of such models, efficient algorithms are required. The efficient use of the modularity in multicellular models by our symbolic algorithm makes it particularly suitable for analyzing cell-cell communication processes together with complex intracellular pathways, a level of detail that would be intractable for enumerative or simpler symbolic algorithms. The utility of this algorithm extends beyond the scope of our iterative improvement approach. The computation of attractors of Boolean networks is, for example, mainly done using enumerative methods. Such methods become intractable even for small models, and since Boolean networks are a particular instance of Qualitative networks, our symbolic algorithm can be directly applied to this problem.

### Modeling approach

In addition to being well suited for our formal verification based iterative improvement approach, using Qualitative network has several benefits compared to using other existing frameworks, such a quantitative models using differential equations and Boolean networks. In this section, we discuss these advantages, and then stress the benefit of clearly separating the modeling of a system using a well defined framework like Qualitative networks from its implementation.

Using discrete qualitative variables sets, allows modeling at a level of abstraction which is similar to the one used in many biological studies. Quantitative methods, such as differential equation-based models, allow continuous and precise modeling of the execution of a system, but require fine-tuning of multiple parameters that are often not easily available for many biological systems. The amount of precision provided by such methods may be unnecessary to analyze a putative model on the basis of experimental results that only provide qualitative levels for the components of the system. The results obtained using Qualitative network models of network motifs that occur frequently, such as the I1-FFL, indicate that the Qualitative networks approach provides a realistic qualitative approximation of the experimentally observed behavior of the system.

The iterative improvement approach used for the construction of a consistent model as well as our analysis algorithms can be applied to Boolean networks. However, extending Boolean networks allows both to create more detailed models in terms of granularity, and to model a richer set of biological interactions thanks to the introduction of the target function. Using more than two levels is necessary if an experimental result cannot be formulated as a requirement over boolean values. This is the case if, for example, the knockout of gene *A *leads to an increase in the level of protein *B*, but the combined knockout of genes *A *and *B *leads to a significantly larger increase. An additional example for which at least three levels are needed is the case when in one experiment the level of a protein decreases, while in another it increases.

The advantages of using Qualitative networks are not limited to the increased expressive power of the specifications. Boolean networks are not sufficient to model some commonly observed patterns. For example, the action of two transcription activators of a gene can be cumulative, whereas Boolean networks only allow modeling the case in which the presence of one transcription factor is sufficient for transcription to happen. Furthermore, while the influence of the I1-FFL motif on the response time can be modeled using Qualitative networks, this is not the case with Boolean networks. In the case of the studied example, the requirement that the presence of *CRP *necessarily leads to the activation of *galETK *implies that, in a Boolean network, the value of *galETK *will necessarily depend directly on the value of *CRP*, and the contribution of *GalS *needs to be ignored. An even simpler example that cannot be reproduced by Boolean networks is negative auto-regulation, where a transcription factor represses its own expression. Negative auto-regulation has been shown to lead to faster response times [43]. In Boolean networks, it is necessary to ignore negative auto regulation, since considering an inhibitory interaction of a component with itself would lead to oscillations.

These examples illustrate the need for extending Boolean networks to the qualitative domain. Depending on the structure of the studied network and the corresponding specifications, Boolean networks may however be sufficient in some cases. Since it is still possible to use the exact same analysis approach, Boolean values should be used in such a situation to reduce the size of the state space. Possible extensions of the Qualitative network framework include the addition of probabilities, resulting in Markov processes, for which formal verification methods exist. However, the necessary data for such models is often not available, and verification of probabilistic systems is computationally harder.

#### Separation between the model and the implementation

We specify the molecular interactions between components by defining a Qualitative network of the studied system. Compared to directly implementing an executable model, this approach allows to clearly separate this specification from the implementation. This allows discussion at the level of the Qualitative network, and thus leads to a improvement process at the level of abstraction often observed in diagrammatic models, which are commonly used by biologists. Furthermore, the clarity and coherence of the model is increased by defining a small set of target function that are consistently reused to model the same kind of interactions. The benefits of a clear separation between the model and its implementation extend beyond Qualitative networks. Using a modular approach based on simple building blocks allows a faster development of coherent executable models. Each type of building block represents a specific kind of biological interaction between components. This approach separates the executable model into two distinct parts: the definition of the interactions between proteins such as activation or inhibition, and the internal implementation of the building blocks. This modular design is particularly useful for fast search of multiple variations in the interaction model. Once the set of basic building blocks is implemented, interactions between biological elements of the model can be changed by replacing one building block by another, and new elements can be added as instances of the appropriate kind of building blocks, all this with minimal changes in the implementation of the model. Changes to the implementation of building blocks only need to be made in one place and can then be reused by all variations of the model. In the case that we want to explore the behavior of the model when we use different ways for representing the interactions between biological elements, it is sufficient to replace one set of basic building blocks by another set of basic building blocks. The clear separation between the interaction model and the implementation is also helpful for assessing the validity of the Qualitative networks approach to modeling biological systems. Rather than having to understand the behavior of every single protein of every variation of the model, our approach allows to first agree on the definition of specific target functions, and then assess the plausibility of a particular Qualitative network, whose representation is close to the common visualization of biochemical pathways. The clear distinction between what we model and how we do it therefore makes the whole approach easier to understand. The clear and concise definition of the behavior of a small set of basic building blocks makes it possible to evaluate (and criticize) the conceptual elements of our approach. Finally, this approach allows to clearly separate methodological issues from issues that are only related to a particular Qualitative network.

### Notions of concurrency

Qualitative networks, like Boolean networks, are synchronous. At each time step, the state of all variables is updated according to the previous state of the system. In this section, we discuss the applicability of other notions of concurrency in the context of Qualitative networks. The variables we use are qualitative representations of the level of protein activity or gene expression. Hence, these variables represent a large population of individual molecules. The interactions between individual molecules are highly stochastic. When using discrete levels of protein activity or gene expression, we consider that the biochemical reactions have a delay. The stochasticity of the individual reactions results in small variations of this delay. The level of all variables is updated according to the previous values. A change therefore takes one time step to propagate from one variable to the next. The time step can thus be considered as being the delay of the biochemical reactions. In case there are major differences between the delays of the different reactions, it is possible to use target functions on the previous states of the systems rather than on the current state. The impact of the small variations of the delay depend on the granularity of the model. The minimal difference between two possible values of a variable is inversely proportional to the granularity of the variable. In a model with a small granularity, variations of the delay would lead to the same value due to rounding. In this case, the synchronous execution is sufficient to reproduce the behavior of the model. As the granularity increases, rounding is no longer sufficient for handling the variability of the delays. In order to model this variability, it is necessary to introduce non-determinism by allowing asynchronous execution of the model. In a totally asynchronous system, a scheduler would choose any subset of the system which would be updated, while the other remain constant. This means that the delay of an interaction is completely independent of the delay of other interactions of the system as well as of the previous delays of the same interaction. This independence is thus an over-approximation of the expected behavior of the system, and is therefore likely to result in unrealistic executions. Therefore, while a synchronous execution is appropriate when the granularity is low, more precise models in terms of granularity call for a better handling of concurrency.

### The state explosion problem

The exhaustive exploration of all possible executions of a system is exponential in the number of variables of the system. This issue is called the *state explosion problem*. For example, in the case of finding all infinitely visited states, the unrestricted number of initial states of a system with *k *variables and granularity *N *is *N*^*k*^. Enumerative methods are practically intractable even for small values of *k*. Furthermore, the complexity of the Notch/Wnt model also caused the direct application of symbolic methods to fail for models of more than three cells. The number of variables of a system not only depends on the size of the studied pathways, but also on the number of cells represented in the model. Hence, the number of initial states is also exponential in the number of cells, which makes multicellular models hard to analyze. In order to be able to analyze larger models, it is therefore necessary to use more elaborate methods that have the potential for circumventing the state explosion problem in specific cases. We provide an illustration of this approach in the symbolic algorithm used for computing the infinitely visited states. We use both the modularity and the specific characteristics of transitions of our model in order to allow interleaving composition and computation of infinitely visited states. The characteristics of our model also make it well suited for the use of partitioned transition relations. We are able to compute the set of infinitely visited states for a 12 cells model, which has 4^144 ^≈ 10^86 ^initial states. Our approach is therefore well suited for analyzing multicellular Qualitative networks. This kind of model is particularly useful for studying pathways that involve inter-cellular communication.

In addition to using the specificity of the model for tailoring the verification algorithm, methods used in software and hardware modification, such as assume-guarantee reasoning [44], or *Counterexample-guided Abstraction Refinement *[45] could also be applied to models of biological systems. Doing so has the potential for making it possible to analyze larger models. In the context of the Notch/Wnt model, this could allow studying multidimensional tissues with larger numbers of cells, and thus the analysis of local effects, such as cell clusters.

## Conclusion

In this work we propose an extension to Boolean networks by allowing variables that represent the level of protein activity or gene expression to range over larger domains, combined with more flexible update options for these values. We show that a Qualitative networks model of a frequent network motif offers a valid qualitative approximation of the experimentally observed behavior of this motif. This framework can be used to model recurrent networks. In addition we use formal verification methods to analyze the steady state behavior of Qualitative networks. Reasoning about sets of states rather than individual states, and using a partition reduction allows us to scale-up to very large models. In particular, Qualitative networks can be used to analyze multi-cellular models, and thus to study pathways that involve cell-cell communication. Similar to Boolean networks, this approach could be useful for the analysis of biological pathways even where quantitative data is missing. We believe that the ability to formally verify a biological model versus the laboratory observations offers great advantages in improving working hypotheses and suggesting new experimental directions that are likely to yield new findings.

## Methods

We use the *Reactive Modules *(RM) formalism [46] to implement executable models based on Qualitative networks. In this section, we first introduce RM, then describe how we make use of its modular structure for this implementation. We describe how specifications are derived from experimental data, and how the implementation of the model is verified against them. Finally, we describe the symbolic algorithm used to compute the infinitely visited states of a Qualitative network.

### Verification of reactive systems

#### Reactive modules

Reactive modules is a modeling language for reactive systems [46]. RM is designed to describe systems which are discrete, deadlock-free, and non-deterministic. The elementary particles in RM are variables. We describe the behavior of variables and the combinations of atoms into modules. Modules can be combined to create more complicated modules (including combinations of several copies of the same module). This process is called *parallel composition*. The variables of a module are defined as either *private, interface *or *external*. The scope of a private variable is limited to its module. An interface variable is *controlled *by an atom of the module and its value can be used by other modules. An external variable is not controlled by any atom in the module. An interface variable of one module can be connected to an external variable of another module or also few other modules. Each variable ranges over a finite set of possible values. An atom describes the possible updates on variables. An atom can be synchronous, meaning that it updates the variables it controls in every step of the system, or asynchronous, meaning that it updates the variables it controls from time to time. An update of a variable may depend on the value of itself as well as the values of other variables. There can also be dependencies between the mutual update of several variables in the same step. In order to compute the future value of a variable it controls, an atom can either *read *the current state of variables or *await *the future state of variables. It is necessary to make sure that the await relation between variables is loop-free. RM enables non-determinism by allowing multiple overlapping update options.

#### Mocha

Mocha [47] is a software tool for the design and analysis of RM. Mocha can simulate a model by following step by step evolution of the variables in the model. Simulations show the sequence of values assumed by variables during the simulation. In simulation of non-deterministic models, the user is expected to choose the next step between different non-deterministic options. The simulation engine can highlight the variable values that lead to the assignment of a certain value. Mocha supports invariant model checking directly (to check that all reachable states satisfy some property that relates to the values of variables in the state), as well as model checking of safety properties using monitors (to check that all executions satisfy some property). Counter-examples are presented as sequences of variable values.

### Modular implementation of Qualitative networks

The implementation of Qualitative networks extends and adapts the modularity of reactive modules to biological models. The list of target functions defines the relation between components by specifying one target function per variable. We separate this model into *building blocks*, where each building block controls one variable. Each building block is an instance of a *basic building block*. For each kind of target function (such as the minimum target function or the activation/inhibition target function), we create one *basic building block*. Note that, since Mocha does not accept parameters, we cannot create a single basic building block for the activation/inhibition target function, and we need to create one for each combination of the parameters *α*_*ij *_used in the model. Instances of the basic building blocks are combined together according to the list of target functions by connecting the output variables of one building block to the input variables of one or several other building blocks. A cell is represented by the parallel composition of several building block instances that hides internal components (makes them private variables) and represents interactions between the cell and the environment as interface and external variables, thus allowing for connection with other cells. This architecture is a consequence of the modular approach of RM, which uses a concept of connection similar to the connections between components in an electrical circuit.

The implementation of each basic building block constitutes one Module, with one or more external variables (corresponding to the input of the target function) and one interface variable (the value of the component controlled by the building block). An additional variable is used to control the behavior of the building block. This variable is only awaited and therefore does not contribute to the size of the state space. The building block is composed of two atoms. The first atom computes the value of the target function of the building block based on the values of the input variables in the current state. This atom is specific to every building block. It is obtained directly from the definition of the corresponding target function, by mapping all possible valuations of the input to the corresponding target value. The second atom implements Equation 2 to compute the value of the output value in the next state of the system based on the target, the current value of the system and the control value. This atom is the same for all building blocks. It makes sure that the output variable increases and decreases by at most one level per step rather than directly jumping from its current state to the target. The control variable allows to specify the initial value or to choose to non-deterministically start from every possible value.

We also introduce the possibility for a building block to determine the initial output value of a building block based on the initial value of its inputs rather than having to set it arbitrarily or non-deterministically. This allows to obtain an initial state of the system in which only a few variables are set and the others are logically derived from them. Furthermore, choosing non-deterministic initial values for a few variables and then deriving the other initial variables leads to significantly less possible initial states than choosing the initial value of every variable non-deterministically, while still exploring the most plausible initial states. In case that there are mutual dependencies between variables (i.e., a component ultimately affects itself in a loop), this approach can be applied only to some of the substances. In such a case, trying to simplify the initial state of all substances would create a loop of dependencies that cannot be solved. It is therefore necessary to choose at least one variable in every loop of the model for which the initial value is set or non-deterministic.

### Formulating specifications

Specifications play a key role in our approach: they are the link between the experimental results and the computational model. A putative model needs to adhere to the specifications in order to be considered as a potentially valid representation of the biological system. If the model does not adhere to a requirement of the specification, then we already know that the model is not able to explain all the existing experimental results, and therefore needs to be improved.

When defining specifications, we start from the description of the experiments and derive safety requirements. The observed result can then be formulated as a predicate over the values of the different components of the system. In the most simple case, the specification can be expressed as a predicate over the current state of the system. We can therefore formulate an invariant which must hold on all states of the system. It is however often necessary to consider that the model starts from an arbitrary initial state and needs some iterations to stabilize. We therefore choose an arbitrary number of steps during which the invariant is not checked. This setting allows to verify if the the wild type (normal) model satisfies certain specifications.

We are also interested in verifying the outcome of experiments that include modifications of the biological model. We first consider under which conditions the experiment has been performed. We consider experimental conditions involving two kinds of manipulations: the over-expression of a gene, in which the level of this gene remains at a *high *level and the knockout of a gene, in which a given gene is modified so that it cannot be transcribed into a protein. In both cases, the influence of the upstream pathways onto the modified variable is ignored. Experiments might involve every combination of these perturbations. In order to be able to model these experiments, we modify the control atom of the basic building blocks by adding two values to the control variable: gene knockout and gene over-expression. When the control variable takes one of these values, the target function is ignored. In the case of gene knockout, the targeted protein is not translated at all, and is not present in the cell. We model this situation by keeping the output value at the lowest possible value when the control variable indicates that knockout has occurred. Gene over expression occurs when the level of a given gene is kept constantly high, and we model it similarly by keeping the output value at the highest possible value. We use a *monitor*, which is an additional module that reads the level of the components and controls the control variable of the individual building blocks. The monitor first lets the model stabilize over a certain number of iterations, then changes the behavior of certain building blocks according to the experiment performed, then allows the model to stabilize again over an arbitrary number of iterations, and then checks if the change induced to the experiment corresponds to the outcome of the experiment. The monitor controls a boolean value, which is *true *until this check is performed, and is then set to *false *if the execution does not reproduce the specification. We can therefore use an invariant which corresponds to the monitor being true for all possible states of the model.

Using monitors, we can therefore verify specifications that are richer than invariants but a subset of general safety properties. The monitor described above allows to verify if all executions of the model satisfy a certain specification. When this is not the case, we are interested in knowing if some execution satisfies the specification. We can do this with monitors, by verifying if there is a trace on which the dual of the specification is not satisfied on all states on which the specification is checked.

We are also interested in extending the notion of delays for stabilization of the model, by only verifying the specification on the infinitely visited states. We consider an execution of the model as a prefix followed by a loop in which the model stays indefinitely unless there is some change in the model. The change triggers a similar behavior, with a second prefix and a second loop. Since Mocha does not provide a direct way of detecting loops, we use an over-approximation of the length of the prefix and the loop as delay for the stabilization. When a contradiction is found, we verify that the trace contains a loop, which means that the contradiction occurred in the loop and is thus valid, else we know that we need to increase our approximation. This solution is not usable when the number of different executions is large. In the section below, we propose a symbolic algorithm which finds all infinitely visited states of the model, and verifies a property only on these states. This method does not require the use of monitors.

### Symbolic computation of all infinitely visited states

We propose a symbolic algorithm for computing all infinitely visited states of a Qualitative network. Infinitely visited states of the model represent the stable states of the biological system, and correspond to the notion of attractors in Boolean networks. Our algorithm builds on the modularity of Qualitative networks. It interleaves the computation of infinitely visited states of parts of the model with the composition of these parts. This process ultimately results in a complete model and the set of infinitely visited states of the model. In this section, we first introduce symbolic methods and the tool used for the implementation of the algorithm, and then describe the algorithm.

#### Symbolic methods

Symbolic methods are used to represent sets of states of a system in a compact way. Rather than enumerating all the states of a set, a symbolic representation uses constraints identifying all states in the set. A symbolic representation may therefore be exponentially more compact than the enumerative representation of the same set. Hence, symbolic algorithms operate on representations of sets rather than considering individual states. Symbolic algorithms are successfully used for model checking systems that have very large state spaces [48].

Mocha offers both enumerative and symbolic methods for verifying invariants. However, in order to find the infinitely visited states of large models, we need an algorithm which is specifically tailored for Qualitative networks. We implement this algorithm using the Relational Manipulation Language. We also need to translate the RM implementation of the model into this language, a process that we do not describe here. We use CrocoPat [49] to execute the algorithm on the translated model. This tool is based on Binary Decision Diagrams (BDDs) [29], a data structure for compact representation of large relations.

#### Algorithm

We want to find the set of all infinitely visited states of a module *M*. A *region σ*_*M *_is a set of states of *M*. The set of all states of *M *is represented by ∑_*M *_and the set of all variables of the module by *X*_*M*_.For a given state *s *of *M *and a subset *V *of *X*_*M*_, we define the projection *s*[*V*] as the restriction of *s *to the variables in *V*. Projection is generalized in the natural way to sets of states. The function *post *(*σ*_*M*_) maps the region *σ*_*M *_to the union of the successors of all states in *σ*_*M*_. This function is constructed according to the definition of the module *M*.

In order to obtain the set of infinitely visited states of *M*, we compute the set of states that is mapped to itself by the *post *function. This set is called the fix point of the function. We use the following algorithm to obtain this fix point:

σM0=ΣM 
 MathType@MTEF@5@5@+=feaafiart1ev1aaatCvAUfKttLearuWrP9MDH5MBPbIqV92AaeXatLxBI9gBaebbnrfifHhDYfgasaacH8akY=wiFfYdH8Gipec8Eeeu0xXdbba9frFj0=OqFfea0dXdd9vqai=hGuQ8kuc9pgc9s8qqaq=dirpe0xb9q8qiLsFr0=vr0=vr0dc8meaabaqaciaacaGaaeqabaqabeGadaaakeaaiiGacqWFdpWCdaqhaaWcbaGaemyta0eabaGaeGimaadaaOGaeyypa0Jaeu4Odm1aaSbaaSqaaiabd2eanbqabaGccqqGGaaiaaa@3568@

*i* := 0

repeat

*i* := *i* + 1

σMi:=post(σMi−1) 
 MathType@MTEF@5@5@+=feaafiart1ev1aaatCvAUfKttLearuWrP9MDH5MBPbIqV92AaeXatLxBI9gBaebbnrfifHhDYfgasaacH8akY=wiFfYdH8Gipec8Eeeu0xXdbba9frFj0=OqFfea0dXdd9vqai=hGuQ8kuc9pgc9s8qqaq=dirpe0xb9q8qiLsFr0=vr0=vr0dc8meaabaqaciaacaGaaeqabaqabeGadaaakeaaiiGacqWFdpWCdaqhaaWcbaGaemyta0eabaGaemyAaKgaaOGaeiOoaOJaeyypa0JaemiCaaNaem4Ba8Maem4CamNaemiDaqNaeiikaGIae83Wdm3aa0baaSqaaiabd2eanbqaaiabdMgaPjabgkHiTiabigdaXaaakiabcMcaPiabbccaGaaa@41A6@

**until **σMi−1=σMi 
 MathType@MTEF@5@5@+=feaafiart1ev1aaatCvAUfKttLearuWrP9MDH5MBPbIqV92AaeXatLxBI9gBaebbnrfifHhDYfgasaacH8akY=wiFfYdH8Gipec8Eeeu0xXdbba9frFj0=OqFfea0dXdd9vqai=hGuQ8kuc9pgc9s8qqaq=dirpe0xb9q8qiLsFr0=vr0=vr0dc8meaabaqaciaacaGaaeqabaqabeGadaaakeaaiiGacqWFdpWCdaqhaaWcbaGaemyta0eabaGaemyAaKMaeyOeI0IaeGymaedaaOGaeyypa0Jae83Wdm3aa0baaSqaaiabd2eanbqaaiabdMgaPbaakiabbccaGaaa@3948@

σMinf:=σMi
 MathType@MTEF@5@5@+=feaafiart1ev1aaatCvAUfKttLearuWrP9MDH5MBPbIqV92AaeXatLxBI9gBaebbnrfifHhDYfgasaacH8akY=wiFfYdH8Gipec8Eeeu0xXdbba9frFj0=OqFfea0dXdd9vqai=hGuQ8kuc9pgc9s8qqaq=dirpe0xb9q8qiLsFr0=vr0=vr0dc8meaabaqaciaacaGaaeqabaqabeGadaaakeaaiiGacqWFdpWCdaqhaaWcbaGaemyta0eabaacbiGae4xAaKMae4NBa4Mae4NzaygaaOGaeiOoaOJaeyypa0Jae83Wdm3aa0baaSqaaiabd2eanbqaaiabdMgaPbaaaaa@3A4C@

The region *σ*^*i *^contains all states that can be visited at time step *i *or later. Therefore the region *σ*^*in f *^contains all states that are infinitely visited. In order to show that this algorithm terminates, it is sufficient to show that σMi⊆σMi−1
 MathType@MTEF@5@5@+=feaafiart1ev1aaatCvAUfKttLearuWrP9MDH5MBPbIqV92AaeXatLxBI9gBaebbnrfifHhDYfgasaacH8akY=wiFfYdH8Gipec8Eeeu0xXdbba9frFj0=OqFfea0dXdd9vqai=hGuQ8kuc9pgc9s8qqaq=dirpe0xb9q8qiLsFr0=vr0=vr0dc8meaabaqaciaacaGaaeqabaqabeGadaaakeaaiiGacqWFdpWCdaqhaaWcbaGaemyta0eabaGaemyAaKgaaOGaeyOHI0Sae83Wdm3aa0baaSqaaiabd2eanbqaaiabdMgaPjabgkHiTiabigdaXaaaaaa@3972@. We can do so by recurrence on σMi
 MathType@MTEF@5@5@+=feaafiart1ev1aaatCvAUfKttLearuWrP9MDH5MBPbIqV92AaeXatLxBI9gBaebbnrfifHhDYfgasaacH8akY=wiFfYdH8Gipec8Eeeu0xXdbba9frFj0=OqFfea0dXdd9vqai=hGuQ8kuc9pgc9s8qqaq=dirpe0xb9q8qiLsFr0=vr0=vr0dc8meaabaqaciaacaGaaeqabaqabeGadaaakeaaiiGacqWFdpWCdaqhaaWcbaGaemyta0eabaGaemyAaKgaaaaa@3121@, using the fact that the *post *function is monotonous: σMi−1⊆σMi−2⇒post(σMi−1)⊆post(σMi−2)⇒σMi⊆σMi−1
 MathType@MTEF@5@5@+=feaafiart1ev1aaatCvAUfKttLearuWrP9MDH5MBPbIqV92AaeXatLxBI9gBaebbnrfifHhDYfgasaacH8akY=wiFfYdH8Gipec8Eeeu0xXdbba9frFj0=OqFfea0dXdd9vqai=hGuQ8kuc9pgc9s8qqaq=dirpe0xb9q8qiLsFr0=vr0=vr0dc8meaabaqaciaacaGaaeqabaqabeGadaaakeaaiiGacqWFdpWCdaqhaaWcbaGaemyta0eabaGaemyAaKMaeyOeI0IaeGymaedaaOGaeyOHI0Sae83Wdm3aa0baaSqaaiabd2eanbqaaiabdMgaPjabgkHiTiabikdaYaaakiabgkDiElabdchaWjabd+gaVjabdohaZjabdsha0jabcIcaOiab=n8aZnaaDaaaleaacqWGnbqtaeaacqWGPbqAcqGHsislcqaIXaqmaaGccqGGPaqkcqGHgksZcqWGWbaCcqWGVbWBcqWGZbWCcqWG0baDcqGGOaakcqWFdpWCdaqhaaWcbaGaemyta0eabaGaemyAaKMaeyOeI0IaeGOmaidaaOGaeiykaKIaeyO0H4Tae83Wdm3aa0baaSqaaiabd2eanbqaaiabdMgaPbaakiabgAOinlab=n8aZnaaDaaaleaacqWGnbqtaeaacqWGPbqAcqGHsislcqaIXaqmaaaaaa@6A36@ and σM1⊆σM0=ΣM
 MathType@MTEF@5@5@+=feaafiart1ev1aaatCvAUfKttLearuWrP9MDH5MBPbIqV92AaeXatLxBI9gBaebbnrfifHhDYfgasaacH8akY=wiFfYdH8Gipec8Eeeu0xXdbba9frFj0=OqFfea0dXdd9vqai=hGuQ8kuc9pgc9s8qqaq=dirpe0xb9q8qiLsFr0=vr0=vr0dc8meaabaqaciaacaGaaeqabaqabeGadaaakeaaiiGacqWFdpWCdaqhaaWcbaGaemyta0eabaGaeGymaedaaOGaeyOHI0Sae83Wdm3aa0baaSqaaiabd2eanbqaaiabicdaWaaakiabg2da9iabfo6atnaaBaaaleaacqWGnbqtaeqaaaaa@3AA0@.

We are interested in knowing if a predicate *p *holds on all infinitely visited states. We define ⟦*p*⟧ ⊆ ∑_*M *_as the set of all states for which the predicate is true. Therefore we can say that the predicate holds on all infinitely visited sets iff σMinf
 MathType@MTEF@5@5@+=feaafiart1ev1aaatCvAUfKttLearuWrP9MDH5MBPbIqV92AaeXatLxBI9gBaebbnrfifHhDYfgasaacH8akY=wiFfYdH8Gipec8Eeeu0xXdbba9frFj0=OqFfea0dXdd9vqai=hGuQ8kuc9pgc9s8qqaq=dirpe0xb9q8qiLsFr0=vr0=vr0dc8meaabaqaciaacaGaaeqabaqabeGadaaakeaaiiGacqWFdpWCdaqhaaWcbaGaemyta0eabaacbiGae4xAaKMae4NBa4Mae4Nzaygaaaaa@33D7@⊆ ⟦*p*⟧. If this is not the case, we are interested in knowing if there is at least one attractor for which the property holds for all states. We modify the algorithm in order to consider only infinitely visited states for which the property holds:

σM,p0=σMinf∩〚p〛 
 MathType@MTEF@5@5@+=feaafiart1ev1aaatCvAUfKttLearuWrP9MDH5MBPbIqV92AaeXatLxBI9gBaebbnrfifHhDYfgasaacH8akY=wiFfYdH8Gipec8Eeeu0xXdbba9frFj0=OqFfea0dXdd9vqai=hGuQ8kuc9pgc9s8qqaq=dirpe0xb9q8qiLsFr0=vr0=vr0dc8meaabaqaciaacaGaaeqabaqabeGadaaakeaaiiGacqWFdpWCdaqhaaWcbaGaemyta0KaeiilaWIaemiCaahabaGaeGimaadaaOGaeyypa0Jae83Wdm3aa0baaSqaaiabd2eanbqaaGqaciab+LgaPjab+5gaUjab+zgaMbaakiabgMIihlablQbmNkabdchaWjablUbmOkabbccaGaaa@420A@

*i* := 0

repeat

*i* := *i* + 1

σM,pi:=post(σM,pi−1)∩〚p〛
 MathType@MTEF@5@5@+=feaafiart1ev1aaatCvAUfKttLearuWrP9MDH5MBPbIqV92AaeXatLxBI9gBaebbnrfifHhDYfgasaacH8akY=wiFfYdH8Gipec8Eeeu0xXdbba9frFj0=OqFfea0dXdd9vqai=hGuQ8kuc9pgc9s8qqaq=dirpe0xb9q8qiLsFr0=vr0=vr0dc8meaabaqaciaacaGaaeqabaqabeGadaaakeaaiiGacqWFdpWCdaqhaaWcbaGaemyta0KaeiilaWIaemiCaahabaGaemyAaKgaaOGaeiOoaOJaeyypa0JaemiCaaNaem4Ba8Maem4CamNaemiDaqNaeiikaGIae83Wdm3aa0baaSqaaiabd2eanjabcYcaSiabdchaWbqaaiabdMgaPjabgkHiTiabigdaXaaakiabcMcaPiabgMIihlablQbmNkabdchaWjablUbmOcaa@4B7E@

**until **σM,pi−1=σM,pi
 MathType@MTEF@5@5@+=feaafiart1ev1aaatCvAUfKttLearuWrP9MDH5MBPbIqV92AaeXatLxBI9gBaebbnrfifHhDYfgasaacH8akY=wiFfYdH8Gipec8Eeeu0xXdbba9frFj0=OqFfea0dXdd9vqai=hGuQ8kuc9pgc9s8qqaq=dirpe0xb9q8qiLsFr0=vr0=vr0dc8meaabaqaciaacaGaaeqabaqabeGadaaakeaaiiGacqWFdpWCdaqhaaWcbaGaemyta0KaeiilaWIaemiCaahabaGaemyAaKMaeyOeI0IaeGymaedaaOGaeyypa0Jae83Wdm3aa0baaSqaaiabd2eanjabcYcaSiabdchaWbqaaiabdMgaPbaaaaa@3D09@

σM,pinf:=σM,pi
 MathType@MTEF@5@5@+=feaafiart1ev1aaatCvAUfKttLearuWrP9MDH5MBPbIqV92AaeXatLxBI9gBaebbnrfifHhDYfgasaacH8akY=wiFfYdH8Gipec8Eeeu0xXdbba9frFj0=OqFfea0dXdd9vqai=hGuQ8kuc9pgc9s8qqaq=dirpe0xb9q8qiLsFr0=vr0=vr0dc8meaabaqaciaacaGaaeqabaqabeGadaaakeaaiiGacqWFdpWCdaqhaaWcbaGaemyta0KaeiilaWIaemiCaahabaacbiGae4xAaKMae4NBa4Mae4NzaygaaOGaeiOoaOJaeyypa0Jae83Wdm3aa0baaSqaaiabd2eanjabcYcaSiabdchaWbqaaiabdMgaPbaaaaa@3EDE@

If σM,pinf
 MathType@MTEF@5@5@+=feaafiart1ev1aaatCvAUfKttLearuWrP9MDH5MBPbIqV92AaeXatLxBI9gBaebbnrfifHhDYfgasaacH8akY=wiFfYdH8Gipec8Eeeu0xXdbba9frFj0=OqFfea0dXdd9vqai=hGuQ8kuc9pgc9s8qqaq=dirpe0xb9q8qiLsFr0=vr0=vr0dc8meaabaqaciaacaGaaeqabaqabeGadaaakeaaiiGacqWFdpWCdaqhaaWcbaGaemyta0KaeiilaWIaemiCaahabaacbiGae4xAaKMae4NBa4Mae4Nzaygaaaaa@3620@ is empty, then there is no attractor on which the property holds for every state. Note that we start from σMinf
 MathType@MTEF@5@5@+=feaafiart1ev1aaatCvAUfKttLearuWrP9MDH5MBPbIqV92AaeXatLxBI9gBaebbnrfifHhDYfgasaacH8akY=wiFfYdH8Gipec8Eeeu0xXdbba9frFj0=OqFfea0dXdd9vqai=hGuQ8kuc9pgc9s8qqaq=dirpe0xb9q8qiLsFr0=vr0=vr0dc8meaabaqaciaacaGaaeqabaqabeGadaaakeaaiiGacqWFdpWCdaqhaaWcbaGaemyta0eabaacbiGae4xAaKMae4NBa4Mae4Nzaygaaaaa@33D7@ since this set has already been computed, but that this fix point can also be used with σM,p0
 MathType@MTEF@5@5@+=feaafiart1ev1aaatCvAUfKttLearuWrP9MDH5MBPbIqV92AaeXatLxBI9gBaebbnrfifHhDYfgasaacH8akY=wiFfYdH8Gipec8Eeeu0xXdbba9frFj0=OqFfea0dXdd9vqai=hGuQ8kuc9pgc9s8qqaq=dirpe0xb9q8qiLsFr0=vr0=vr0dc8meaabaqaciaacaGaaeqabaqabeGadaaakeaaiiGacqWFdpWCdaqhaaWcbaGaemyta0KaeiilaWIaemiCaahabaGaeGimaadaaaaa@32FD@ = ⟦*p*⟧. The kind of analysis we are interested in does not need explicit computation of the individual attractors. Obtaining the explicit trace of each attractor would need to be done in an enumerative way, and thus requires *O *(|σMinf
 MathType@MTEF@5@5@+=feaafiart1ev1aaatCvAUfKttLearuWrP9MDH5MBPbIqV92AaeXatLxBI9gBaebbnrfifHhDYfgasaacH8akY=wiFfYdH8Gipec8Eeeu0xXdbba9frFj0=OqFfea0dXdd9vqai=hGuQ8kuc9pgc9s8qqaq=dirpe0xb9q8qiLsFr0=vr0=vr0dc8meaabaqaciaacaGaaeqabaqabeGadaaakeaaiiGacqWFdpWCdaqhaaWcbaGaemyta0eabaacbiGae4xAaKMae4NBa4Mae4Nzaygaaaaa@33D7@|) steps (for a deterministic Qualitative network).

The general algorithm described above can be applied to every kind of module. We use a more specific algorithm tailored for Qualitative networks. Each module consists of an atom computing the target function and a controller atom which enforces the transition rules specified in Equation (2). Rather than directly computing the infinitely visited region of a composed module *M *= *M*_1_||*M*_2_, we first compute the infinitely visited region of each module separately, and then do the parallel composition of them. The separate modules might have external variables, whose behavior is not specified. Rather than allowing them to have any random behavior, we enforce the transition rules of Equation (2). The set of possible behaviors of an external variable is therefore a superset of the behavior of any building block. Using the algorithm above, we compute the infinitely visited region σM1inf
 MathType@MTEF@5@5@+=feaafiart1ev1aaatCvAUfKttLearuWrP9MDH5MBPbIqV92AaeXatLxBI9gBaebbnrfifHhDYfgasaacH8akY=wiFfYdH8Gipec8Eeeu0xXdbba9frFj0=OqFfea0dXdd9vqai=hGuQ8kuc9pgc9s8qqaq=dirpe0xb9q8qiLsFr0=vr0=vr0dc8meaabaqaciaacaGaaeqabaqabeGadaaakeaaiiGacqWFdpWCdaqhaaWcbaGaemyta00aaSbaaWqaaiabigdaXaqabaaaleaaieGacqGFPbqAcqGFUbGBcqGFMbGzaaaaaa@34FF@ of *M*_1_. We consider the projection σMinf[XM1]
 MathType@MTEF@5@5@+=feaafiart1ev1aaatCvAUfKttLearuWrP9MDH5MBPbIqV92AaeXatLxBI9gBaebbnrfifHhDYfgasaacH8akY=wiFfYdH8Gipec8Eeeu0xXdbba9frFj0=OqFfea0dXdd9vqai=hGuQ8kuc9pgc9s8qqaq=dirpe0xb9q8qiLsFr0=vr0=vr0dc8meaabaqaciaacaGaaeqabaqabeGadaaakeaaiiGacqWFdpWCdaqhaaWcbaGaemyta0eabaacbiGae4xAaKMae4NBa4Mae4NzaygaaOGaei4waSLaemiwaG1aaSbaaSqaaiabd2eannaaBaaameaacqaIXaqmaeqaaaWcbeaakiabc2faDbaa@3A1B@ of the infinitely visited states of *M *onto the variables of *M*_1_. Since the set of behaviors of the non-deterministic building block are a superset of the behavior of any other building block we have that σMinf[XM1]
 MathType@MTEF@5@5@+=feaafiart1ev1aaatCvAUfKttLearuWrP9MDH5MBPbIqV92AaeXatLxBI9gBaebbnrfifHhDYfgasaacH8akY=wiFfYdH8Gipec8Eeeu0xXdbba9frFj0=OqFfea0dXdd9vqai=hGuQ8kuc9pgc9s8qqaq=dirpe0xb9q8qiLsFr0=vr0=vr0dc8meaabaqaciaacaGaaeqabaqabeGadaaakeaaiiGacqWFdpWCdaqhaaWcbaGaemyta0eabaacbiGae4xAaKMae4NBa4Mae4NzaygaaOGaei4waSLaemiwaG1aaSbaaSqaaiabd2eannaaBaaameaacqaIXaqmaeqaaaWcbeaakiabc2faDbaa@3A1B@ is included in σM1inf
 MathType@MTEF@5@5@+=feaafiart1ev1aaatCvAUfKttLearuWrP9MDH5MBPbIqV92AaeXatLxBI9gBaebbnrfifHhDYfgasaacH8akY=wiFfYdH8Gipec8Eeeu0xXdbba9frFj0=OqFfea0dXdd9vqai=hGuQ8kuc9pgc9s8qqaq=dirpe0xb9q8qiLsFr0=vr0=vr0dc8meaabaqaciaacaGaaeqabaqabeGadaaakeaaiiGacqWFdpWCdaqhaaWcbaGaemyta00aaSbaaWqaaiabigdaXaqabaaaleaaieGacqGFPbqAcqGFUbGBcqGFMbGzaaaaaa@34FF@. Similarly, we compute the infinitely visited region of *M*_2_, σM2inf
 MathType@MTEF@5@5@+=feaafiart1ev1aaatCvAUfKttLearuWrP9MDH5MBPbIqV92AaeXatLxBI9gBaebbnrfifHhDYfgasaacH8akY=wiFfYdH8Gipec8Eeeu0xXdbba9frFj0=OqFfea0dXdd9vqai=hGuQ8kuc9pgc9s8qqaq=dirpe0xb9q8qiLsFr0=vr0=vr0dc8meaabaqaciaacaGaaeqabaqabeGadaaakeaaiiGacqWFdpWCdaqhaaWcbaGaemyta00aaSbaaWqaaiabikdaYaqabaaaleaaieGacqGFPbqAcqGFUbGBcqGFMbGzaaaaaa@3501@ we can now compute a superset of σMinf:σMinf⊆{s∈ΣM|s[XM1]∈σM1inf and s[XM2]∈σM2inf}
 MathType@MTEF@5@5@+=feaafiart1ev1aaatCvAUfKttLearuWrP9MDH5MBPbIqV92AaeXatLxBI9gBaebbnrfifHhDYfgasaacH8akY=wiFfYdH8Gipec8Eeeu0xXdbba9frFj0=OqFfea0dXdd9vqai=hGuQ8kuc9pgc9s8qqaq=dirpe0xb9q8qiLsFr0=vr0=vr0dc8meaabaqaciaacaGaaeqabaqabeGadaaakeaaiiGacqWFdpWCdaqhaaWcbaGaemyta0eabaacbiGae4xAaKMae4NBa4Mae4NzaygaaOGaeiOoaOJae83Wdm3aa0baaSqaaiabd2eanbqaaiab+LgaPjab+5gaUjab+zgaMbaakiabgAOinpaacmaabaGaem4CamNaeyicI4Saeu4Odm1aaSbaaSqaaiabd2eanbqabaGccqGG8baFcqWGZbWCcqGGBbWwcqWGybawdaWgaaWcbaGaemyta00aaSbaaWqaaiabigdaXaqabaaaleqaaOGaeiyxa0LaeyicI4Sae83Wdm3aa0baaSqaaiabd2eannaaBaaameaacqaIXaqmaeqaaaWcbaGae4xAaKMae4NBa4Mae4NzaygaaOGaeeyyaeMaeeOBa4MaeeizaqMaeeiiaaIaem4CamNaei4waSLaemiwaG1aaSbaaSqaaiabd2eannaaBaaameaacqaIYaGmaeqaaaWcbeaakiabc2faDjabgIGiolab=n8aZnaaDaaaleaacqWGnbqtdaWgaaadbaGaeGOmaidabeaaaSqaaiab+LgaPjab+5gaUjab+zgaMbaaaOGaay5Eaiaaw2haaaaa@6F29@. We use this smaller set as initial set σM0
 MathType@MTEF@5@5@+=feaafiart1ev1aaatCvAUfKttLearuWrP9MDH5MBPbIqV92AaeXatLxBI9gBaebbnrfifHhDYfgasaacH8akY=wiFfYdH8Gipec8Eeeu0xXdbba9frFj0=OqFfea0dXdd9vqai=hGuQ8kuc9pgc9s8qqaq=dirpe0xb9q8qiLsFr0=vr0=vr0dc8meaabaqaciaacaGaaeqabaqabeGadaaakeaaiiGacqWFdpWCdaqhaaWcbaGaemyta0eabaGaeGimaadaaaaa@30B4@ for the computation of the infinitely visited states of *M*. By repeating this iteratively we can handle larger compositions.

When applying this method to a multicellular model, we use *partitioned transition relations *[30]. Rather than defining a single *post *function for the whole model, we define one function per cell, and apply them successively.

### Setting of the performance analysis

The performance tests are performed on a 3 GHz Intel Xeon computer running Linux Fedora Core 3 (Kernel version 2.6.11). The CrocoPat tool can use up to 3 GB of memory.

## Authors' contributions

MS carried out the modeling work, conceived the idea of the Qualitative network framework, and performed the analysis of the model. JF conceived the study and participated in its design. TAH participated in the design of this study. All authors collaborated for the elaboration of the manuscript, read, and approved the final manuscript.

## Figures and Tables

**Table 1:** Execution times for variations of the Notch/Wnt model

Size	Pattern	Time	Initial states	Inf. visited states
3	OMH	33 sec.	4^36 ^≈ 10^21^	1
4	OMHH	4 min. 27 sec.	4^48 ^≈ 10^28^	256
5	OMHHH	21 min.	4^60 ^≈ 10^36^	6561
6	OOMHHH	24 min.	4^72 ^≈ 10^43^	6561
7	OOOMHHH	26 min.	4^84 ^≈ 10^50^	6561
8	OOOLMHHH	63 min.	4^96 ^≈ 10^57^	256
9	OOOLMHHHH	171 min.	4^108 ^≈ 10^65^	256
10	OOOOLMHHHH	181 min.	4^120 ^≈ 10^72^	256
11	OOOOLMHHHHH	513 min.	4^132 ^≈ 10^79^	256
12	OOOOOLMHHHHH	543 min.	4^144 ^≈ 10^86^	256

This table indicates the execution time for various sizes of the model. The patterns indicate the fixed amount of *Notch *receptor chosen for each cell of the model. The letters *O*, *L*, *M *and *H *represents a cell in which the amount of Notch receptor is set to respectively *off, low, medium *and *high*. The number of initial states (which is equal to the number of states of the model) and the number of infinitely visited states are also indicated.

**Table 2:** Size of infinitely visited state sets for partial models

Size	Before	After	BDD Nodes
1	1.4 · 10^6^	3424	427
2	2.9 · 10^8^	3424	451
3	2.9 · 10^8^	3424	475
4	2.9 · 10^8^	3424	499
5	4.5 · 10^8^	1.3 · 10^6^	1189
6	2.5 · 10^11^	5.2 · 10^6^	23092
7	1.0 · 10^12^	7.2 · 10^8^	70626
8	1.4 · 10^13^	5.6 · 10^8^	146169
9	1.1 · 10^14^	5.5 · 10^9^	253558
10	6.1 · 10^13^	255	8830

This table describes the execution of the algorithm on the 10 cells Notch/Wnt model. For each partial model, the number of potentially infinitely visited states before the execution of the algorithm and the number of infinitely visited states found by the algorithm are indicated. The size of the symbolic representation of the infinitely visited states of the partition is indicated in terms of number of BDD nodes.
